# A Novel Fibroblast Activation Protein‐Based Algorithm to Assess Fibrosis in Metabolic Dysfunction–Associated Steatotic Liver Disease

**DOI:** 10.1111/jgh.70294

**Published:** 2026-02-16

**Authors:** Ziqi V. Wang, Badwi B. Boumelhem, Torsten Pennell, William W. Bachovchin, Jack Hung‐Sen Lai, Sarah E. Poplawski, Harsha Chandraratna, Pieter Van Der Veken, Kate Brewer, Diana Julie Leeming, Geraldine Ooi, Jacob George, Mohammed Eslam, Leon A. Adams, Hui Emma Zhang, Geoffrey W. McCaughan, Avik Majumdar, Mark D. Gorrell

**Affiliations:** ^1^ Liver Enzymes in Metabolism and Inflammation, Centenary Institute, Faculty of Medicine and Health The University of Sydney Sydney New South Wales Australia; ^2^ Department of Developmental, Molecular and Chemical Biology, Graduate School of Biomedical Sciences Tufts University Boston Massachusetts USA; ^3^ Obesity Surgery WA Booragoon Western Australia Australia; ^4^ Laboratory of Medicinal Chemistry, Department of Pharmaceutical Sciences University of Antwerp Wilrijk Belgium; ^5^ Nordic Bioscience A/S Herlev Denmark; ^6^ Department of Surgery Monash University Melbourne Victoria Australia; ^7^ Storr Liver Centre, The Westmead Institute for Medical Research Westmead Hospital and the University of Sydney Westmead New South Wales Australia; ^8^ Medical School, Faculty of Medicine and Health Sciences The University of Western Australia Perth Western Australia Australia; ^9^ AW Morrow Gastroenterology and Liver Centre Royal Prince Alfred Hospital Camperdown New South Wales Australia; ^10^ Department of Gastroenterology Austin Hospital Melbourne Victoria Australia; ^11^ The Charles Perkins Centre The University of Sydney Sydney New South Wales Australia

**Keywords:** FAP Index, FIB‐4, fibroblast activation protein, fibrosis, noninvasive test

## Abstract

**Background:**

Noninvasive fibrosis testing is crucial for metabolic dysfunction–associated steatotic liver disease (MASLD) management. This study evaluated a marker of activated mesenchymal fibrogenic cells, circulating fibroblast activation protein (cFAP), in a novel diagnostic algorithm, FAP Index, for patients with MASLD.

**Methods:**

Two retrospective cohorts recruited from tertiary hepatology clinics were studied as training (*n* = 160) and external validation cohorts (*n* = 332), with prevalence of histologic advanced fibrosis (F3–F4) of 20% and 11%, respectively. cFAP was measured using our rapid single‐step FAP‐specific microplate enzyme assay. A predictive model, FAP Index, containing age, type 2 diabetes, alanine transaminase, and ordinal cFAP was developed using logistic regression; then, its diagnostic accuracy was evaluated.

**Results:**

FAP Index AUROC for advanced fibrosis was 0.875 (95% CI: 0.813–0.938) in the training cohort and 0.841 (95% CI: 0.776–0.906) in the validation cohort. Low cutoff 0.157 (sensitivity 84.3%, negative predictive value 95%) and high cutoff 0.695 (specificity 99.2%, positive predictive value 92.9%) values excluded and predicted advanced fibrosis, respectively. FAP Index following FIB‐4 reduced the frequency of indeterminate results by more than one‐third compared to FIB‐4 alone. FAP Index following NFS (NAFLD Fibrosis Score) reduced the frequency of indeterminate results by ~70% compared to NFS alone.

**Conclusion:**

Without a need for elastography, applying FAP Index following FIB‐4 or NFS can facilitate accurate risk stratification of patients by greatly reducing the frequency of indeterminate results compared to FIB‐4 or NFS alone, without compromising negative predictive value. Thus, FAP Index is a novel, fibrogenesis‐relevant, rapid, robust diagnostic tool suited to increasing the efficiency of liver fibrosis triage in primary care.

## Graphical abstract

To improve triage for liver fibrosis in MASLD primary care and where elastography is unavailable, a novel algorithm, FAP Index, that requires one fibrogenesis‐relevant, rapid enzyme assay, was devised and validated. FAP Index increased advanced fibrosis risk stratification accuracy by greatly reducing the frequency of indeterminate results compared to FIB‐4 or NFS alone.

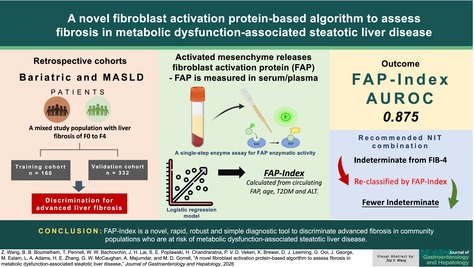

**Abbreviations**
ALP: alkaline phosphatase
ALT: alanine transaminase
AMC: amino‐4‐methylcoumarin
AST: aspartate transaminase
AUROC: area under the receiver operating characteristics curve
BMI: body mass index
cFAP: circulating fibroblast activation protein
FAP: fibroblast activation protein
FGF‐21: fibroblast growth factor 21
GGT: gamma‐glutamyl transferase
HSC: hepatic stellate cell
IQR: interquartile range
MASLD: metabolic dysfunction–associated steatotic liver disease
NAFLD: nonalcoholic fatty liver disease
NFS: NAFLD Fibrosis Score
NIT: noninvasive test
NPV: negative predictive value
PLT: platelets
PPV: positive predictive value
ROC: receiver operating characteristics curve
T2DM: type 2 diabetes mellitus

## 1 Introduction

Metabolic dysfunction–associated steatotic liver disease (MASLD) has become increasingly common, now affecting one quarter of adults [1, 2, 3]. MASLD pathogenesis encompasses lipid dysregulation, glucose dysregulation, gut microbiome alterations, and genetic variants [4] that promote chronic inflammation that can lead to hepatic fibrosis. Fibrosis is the most potent driver, along with obesity, type 2 diabetes mellitus (T2DM), and insulin resistance, toward end‐stage liver disease and hepatocellular carcinoma [2, 4, 5, 6]. The risk of liver‐related morbidity and mortality increases in parallel with progression into advanced hepatic fibrosis and cirrhosis [3, 7]. Therefore, accurately identifying those at risk of advanced fibrosis in the context of increasing MASLD prevalence is an urgent clinical need.

For liver fibrosis assessment, elastography is increasingly preferred over liver biopsy but is not universally accessible in general health practice. Therefore, noninvasive blood tests (NITs) are favored for the diagnosis of advanced liver fibrosis. In MASLD, the two most commonly used models are NAFLD fibrosis score (NFS) [8] and Fibrosis Index 4 (FIB‐4) [9]. These NITs, particularly FIB‐4, are recommended for advanced fibrosis screening [10], but greater accuracy is essential because many indeterminate results are produced [11, 12]. Furthermore, serum tests should ideally include a direct fibrogenesis marker, which FIB‐4 lacks, because a direct marker of fibrogenesis may be reasonably expected to improve diagnostic accuracy. Clinical consensus guidelines increasingly recommend step‐wise strategies for NITs, including serum‐based NITs directed to liver fibrosis, followed by elastography, for advanced fibrosis screening [10, 13, 14]. However, elastography is largely based within hospital systems and is not freely available in the community. Ideally, community‐oriented NITs would rely only on serum assays together with patient demographics as the first triage step for patients with MASLD. Current alternate serum NITs such as ELF or Hepascore include multiple assays that are not routinely available in pathology laboratories, also increasing costs.

Fibroblast activation protein alpha (FAP) is a cell surface and soluble glycoprotein with a unique catalytic activity and cellular expression profile that closely links FAP with fibrogenic cells including myofibroblasts and extracellular matrix proteins [15, 16, 17, 18, 19, 20]. Recent molecular imaging has confirmed the strong quantitative association of intrahepatic FAP with liver fibrosis severity [21] [22, 23]. Circulating FAP (cFAP) enzyme activity [24, 25, 26] increases with advanced fibrosis (F3–F4) [25, 27] and decreases following liver transplantation [26]. Thus, cFAP is a candidate serum biomarker for liver fibrosis that is directly linked with fibrogenesis. The resource use and potential costs of using our FAP assay are modest, similar to measuring ALT [24, 25, 26, 27].

In this study, we developed a NIT‐based fibrosis algorithm, FAP Index, that incorporates our cFAP quantitation to discriminate advanced fibrosis. Moreover, we validated the performance of this algorithm in a MASLD cohort that mimics the prevalence of advanced fibrosis in the community. We showed that applying FAP Index following FIB‐4 or NFS can increase the accuracy of risk stratification of patients by greatly reducing the frequency of indeterminate results compared to FIB‐4 or NFS alone, without compromising negative prediction value (NPV). Thus, FAP Index together with FIB‐4 is a new potential simple NIT‐based approach that could facilitate triaging of patients with MASLD without the initial need for transient elastography.

## 2 Methods

### 2.1 Study Cohorts

The two retrospective study cohorts met MASLD criteria [28]: A training cohort (*n* = 160; 20% advanced fibrosis) [29] and a validation cohort (*n* = 332) [30, 31] that replicated the 10%–15% prevalence of advanced fibrosis in at‐risk MASLD populations [32, 33]. Alcohol‐related (daily mean intakes 30 g for men, 20 g for women), viral hepatitis, and nonmetabolic etiologies were excluded. Ethics approvals (Supplement S1.1) and governance guidelines were adhered to. Routine clinical biochemistry and liver biopsies were completed at the original site of admission based on standardized clinical guidelines and scoring systems (Supplement S1.2).

### 2.2 Quantitative FAP Enzyme Activity Assay

Quantifying cFAP enzyme activity has been described [24, 25, 26]. Microplate wells contained 5 μL of 1:5 diluted serum in 70 μL Tris (10 mM)/EDTA (1 mM) pH 7.4 (TE buffer). Fluorescent product was measured for 1 h at 37°C, after adding 25 μL 150 μM 3144‐AMC (7‐amino‐4‐methylcoumarin) substrate, in a PolarStar plate reader (BMG Labtech, Ortenberg, Germany) at excitation 355 nm, emission 450 nm. AMC at 0–600 pmol in 100 μL TE formed a standard curve. Methods of verifying the specificity of 3144‐AMC for FAP, using pure enzymes and FAP knockout mice [24, 34, 35, 36] are in Supplement S1.3.

### 2.3 Statistical Analyses

The presence of advanced fibrosis (F3–F4) was a binary parameter in this study. Numeric data are presented as median with interquartile range (IQR) due to nonnormality. Missing data triggered exclusion. Mann–Whitney *U* or one‐way ANOVA was performed for group comparisons. Statistics used R (v4.5.1) and GraphPad Prism (GraphPad, v9.4.1), with α value 0.05 for statistical significance. Pearson's correlation assessed linear associations. Univariable logistic regression was performed for dichotomous or ordinal versus continuous variables [37], and chi‐square test for categorical variables. cFAP was transformed into an ordinal variable due to extreme outliers (Supplementary Figure 1A,B), with 0 = *low*, 1 = *middle*, 2 = *high*, using cutoffs 730 [25] and 1580 pmol AMC/min/mL. That high cutoff optimized positive predictive value (PPV) for advanced fibrosis.

A logistic predictive model incorporating cFAP was developed following published methods [37]. Variables with *p* < 0.1 were included from univariable analysis, and then the model, termed FAP Index, was derived by backward elimination. The Hosmer–Lemeshow test assessed goodness of fit. Area under the receiver operating characteristic curves (AUROC) was calculated [38] and compared by DeLong test. Dual cutoffs were chosen to optimize sensitivity, specificity, and percentage indeterminate. Principal component analysis (PCA) assessed contributions to FAP Index by individual variables. Validation included individuals with complete data, so individuals who lacked FAP Index, FIB‐4, NFS, or biopsy score were excluded from validation analyses. FAP Index was compared with FIB‐4 (5) and NFS (4) in subcohorts of 87 (training) and 316 (validation) patients.

## 3 Results

### 3.1 Clinical Characteristics

Clinical characteristics differed between cohorts regarding only age, prevalence of fibrosis stages, and cFAP abundance (Table 1 and Supplementary Table 1). Advanced fibrosis was more prevalent in the training (20.3%) than validation (11.4%) cohort. cFAP was more abundant in the validation than training cohort (1290, IQR = 555.9 vs. 995.7, IQR = 579.3 pmol AMC/min/L; *p* < 0.0001). In both cohorts, cFAP was associated with HOMA2‐IR, insulin, and all three liver transaminases (*p* < 0.05), but not T2DM (Supplementary Table 2).

**TABLE 1:** Baseline cohort characteristics.

	Training cohort (*n* = 160)	Validation cohort (*n* = 332)	*p* ^a^
Age (years)	52 (18.25)	49 (19)	0.02
Gender (male)	58 (36%)	97 (29.2%)	0.14
Diabetes (1)^b^	57 (36%)	98 (29.6%)	0.21
BMI (kg/m^2^)	38.19 (12.05)	38.6 (14.2)	0.89
ALT (U/L)	40 (42.25)	44 (45)	0.28
AST (U/L)	32.5 (20)	33 (26.3)	0.87
GGT (U/L)	40 (60.25)	42 (70.3)	0.87
PLTs (×10^9^/L)	225 (94)	239 (88.3)	0.11
ALP (U/L)	81 (35.5)	Nd	Nd
Insulin (mU/L)	11 (15)	10 (11)	0.42
HOMA2_IR	1.48 (2.07)	1.37 (1.46)	0.46
Fibrosis staging			0.02
F0	81 (51.3%)	180 (54.2%)	
F1	35 (22.2%)	84 (25.3%)	
F2	10 (6.3%)	30 (9%)	
F3	17 (10.8%)	29 (8.7%)	
F4	15 (9.5%)	9 (2.7%)	
Advanced fibrosis	32 (20.3%)	38 (11.4%)	0.01
cFAP (pmol AMC/min/L)	995.74 (579.3)	1290 (555.9)	< 0.0001
cFAP ordinal			< 0.0001
Level 0	38 (23.8%)	15 (4.5%)	
Level 1	94 (58.8%)	228 (68.7%)	
Level 2	28 (17.5%)	89 (26.8%)	

*Note:* Median (IQR) for continuous variables. Prevalence (%) for categorical variables.

^a^ Mann–Whitney *U* test for comparing cohorts.

^b^ T2DM scored 1.

Abbreviations: ALP, alkaline phosphatase; ALT, alanine transaminase; AST, aspartate transaminase; BMI, body mass index; GGT, gamma‐glutamyl transferase; PLT, platelets.

### 3.2 The FAP Index Algorithm

cFAP levels correlated with fibrosis stage (Supplementary Table 2, Supplementary Figure 2B). Moreover, cFAP was significantly greater for patients with F3–F4 than F0 (training cohort *p* < 0.0001; Supplementary Figure 2A,B), consistent with the ability for ruling in or out advanced fibrosis.

Stepwise, backward elimination in multivariable logistic regression yielded age, T2DM, ALT, and cFAP as significantly associated with and thus predictors of advanced fibrosis (Table 2). Therefore, with a suitable Hosmer–Lemeshow test χ^2^ of 3.066 (*p* = 0.93), the FAP Index formula was as follows:

$$\begin{array}{c} \text{Index Score}=-9.499+0.101\times \mathrm{Age}+1.533\times T2\mathrm{DM}\;\left(0\;\text{or}\;1\right)\\ \;+0.009\times \mathrm{ALT}+1.158\times \text{ordinal}\_\text{cFAP}\;\left(0,1,\text{or}\;2\right) \end{array}$$

$$\mathrm{FAP}\;\text{Index}=\frac{\exp\left(\text{Index Score}\right)}{1+\exp\left(\text{Index Score}\right)}$$

**TABLE 2:** Association between each clinical parameter^a^ and advanced fibrosis in the training cohort. Univariate logistic regression^b^ chi‐square test.

	Univariate association analysis			Multivariate regression model		
Parameter	Odds ratio	95% CI	*p*	Odds ratio	95% CI	*p*
Age ^a^	1.11	1.05–1.16	< 0.001	1.11	1.05–1.17	< 0.0001
Gender (male) ^b^	1.45	0.66–3.19	0.36			
Diabetes (yes/no) ^b^	6.92	2.91–16.43	< 0.001	4.58	1.68–12.48	< 0.001
BMI ^a^	0.96	0.91–1.01	0.16			
ALT ^a^	1.01	1–1.01	0.01	1.01	1.00–1.02	0.02
AST ^a^	1.02	1.01–1.03	0.01			
GGT ^a^	1.005	1–1.01	< 0.001			
PLT (×10^9^/L) ^a^	0.99	0.98–0.99	< 0.001			
ALP ^a^	1.02	1.01–1.03	< 0.001			
Insulin (mU/L) ^a^	1.03	1.01–1.06	0.01			
cFAP (pmol AMC/min/L) ^a^	1.002	1.0–1.0	< 0.001			
Ordinal FAP ^b^			< 0.001			0.002
0	—	—		—	—	
1	2.27	0.62–8.36		2.42	0.58–10.11	
2	11.67	2.9–46.96		9.24	1.86–46.01	

Abbreviation: AMC, amino‐4‐methylcoumarin.

**FIGURE 1:**
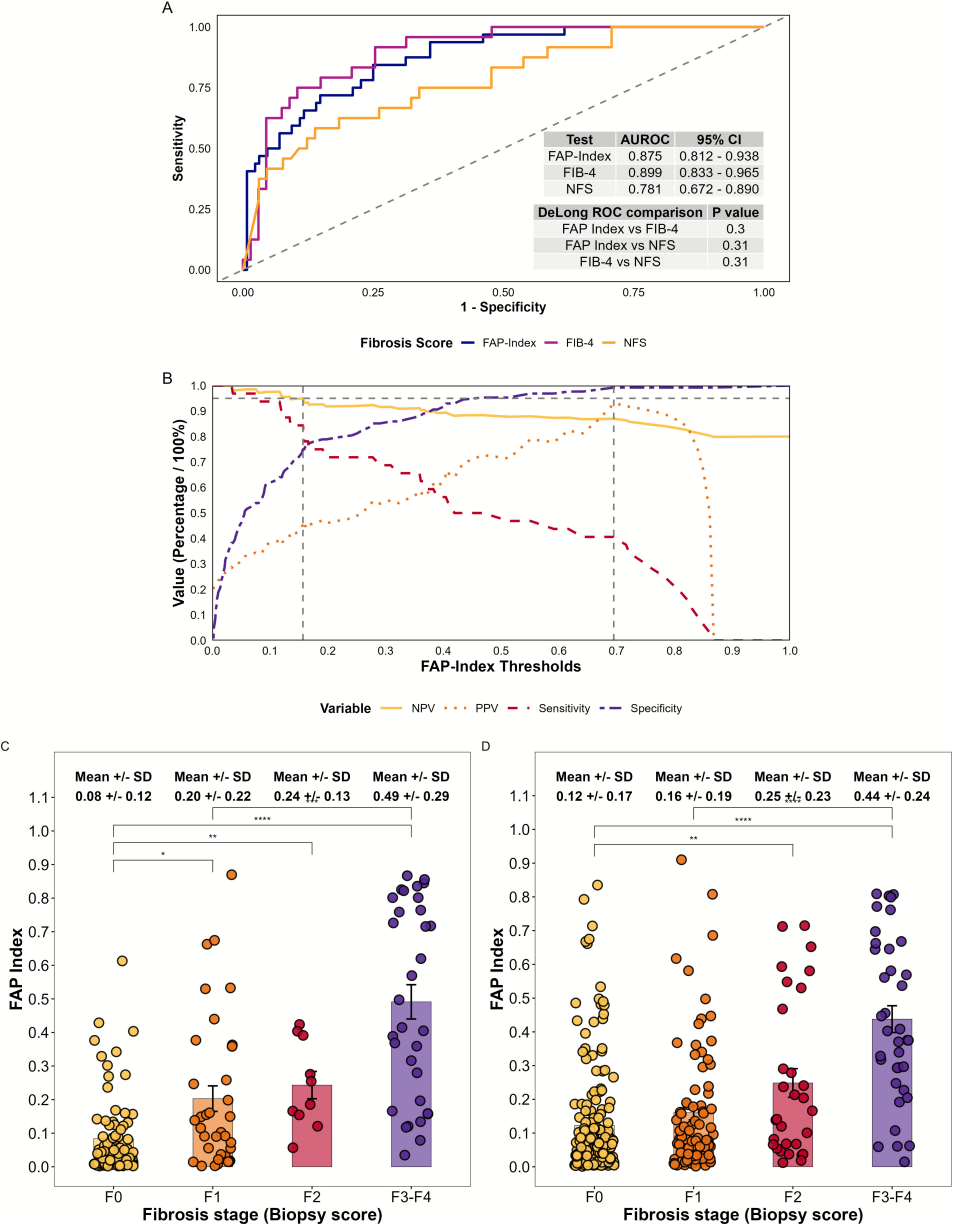
(A) Training cohort FAP Index, FIB‐4, and NFS AUROCs were comparable, by DeLong test. (B) FAP Index diagnostic performance matrices (specificity, sensitivity, NPV, and PPV) over a continuum of thresholds. (C, D) Associations of FAP Index with fibrosis staging in training (C, *n* = 160) and validation (D, *n* = 332) cohorts. Dot plot with mean ± SEM, One‐way ANOVA with Tukey's post hoc test: *p < 0.05, **p < 0.01, ***p < 0.001, ****p < 0.0001.

The FAP Index AUROC for predicting advanced fibrosis was 0.875 (training cohort; 95% CI: 0.812–0.938; Figure 1A). ROC‐derived dual cutoffs were applied to optimize ruling in and out advanced fibrosis, yielding low cutoff 0.157 (sensitivity = 0.84, specificity = 0.75) and high cutoff 0.695 (sensitivity = 0.41, specificity = 0.99). Thus, 0.157 < FAP Index < 0.695 was indeterminate, which maintained NPV at 95% and maximized PPV (Figure 1B), sensitivity, and specificity. Examining thresholds using PCA supported these cutoffs choices (Supplement 2.1). Moreover, compared to cFAP, FAP Index more closely associated with fibrosis severity (Figure 1C,D) and increased differentiation across fibrosis stages, comparable with FIB‐4 (Supplementary Figure 2C,D). FAP Index also demonstrated significant positive correlations with both FIB‐4 and NFS (Supplemental S2.4).

**FIGURE 2:**
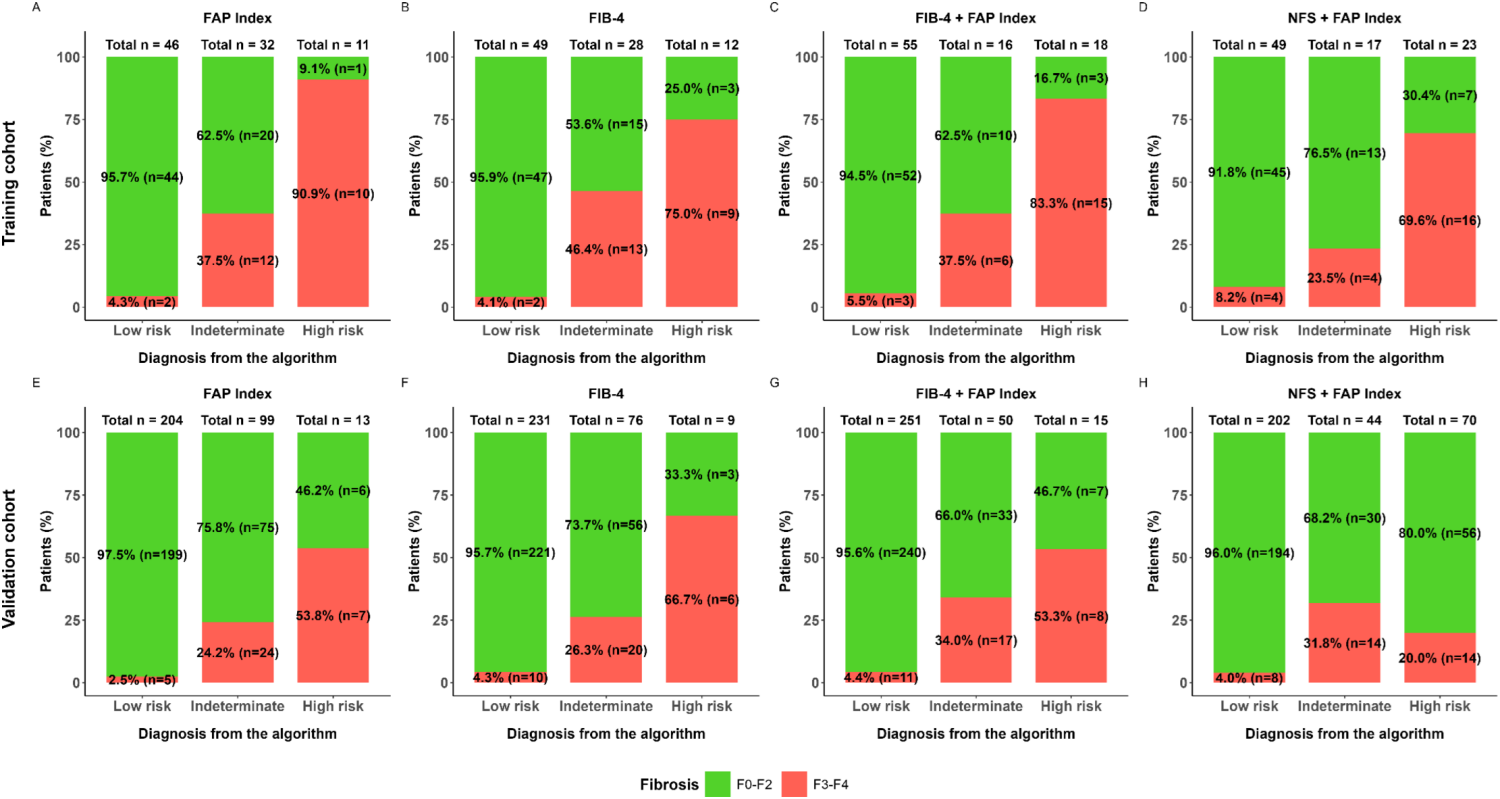
Fibrosis classification accuracy. Stratification for the risk of advanced fibrosis using either single or paired‐sequential application of FAP Index, FIB‐4, FIB‐4/FAP Index, and NFS/FAP Index in the training (A–D) and validation (E–H) cohorts. Colors represent F0–F2 (green) and F3–F4 (red) biopsy‐derived fibrosis score.

The validation cohort FAP Index AUROC for predicting advanced fibrosis was 0.841 (Supplementary Figure 5), with specificity 97%, NPV 97%, sensitivity 58%, and 95% accuracy, with only 31% indeterminate (Figure 2E–F, Supplementary Figure 6F, Table 3).

**TABLE 3:** Summary of classification analyses of outcomes from each test and combination of tests applied to the training subcohort (*n* = 87) and validation cohort (*n* = 316), as referenced to biopsy confirmed advanced fibrosis (F3–F4), and reported for NPV, PPV, proportion of indeterminate, sensitivity, specificity and accuracy.

	Diagnostic matrices					
	Training cohort					
Test applied	NPV	PPV	% Indeterminate	Sensitivity^a^	Specificity^a^	Accuracy
FAP Index	95.7%	90.9%	36.0%	83.3%	97.8%	94.7%
FIB‐4	95.9%	75.0%	31.5%	81.8%	94.0%	91.8%
NFS	92.9%	64.7%	49.4%	84.6%	81.2%	82.2%
FAP Index then FIB‐4	93.0%	87.5%	18.0%	77.8%	96.4%	91.8%
FAP Index then NFS	94.3%	89.5%	19.1%	85.0%	96.2%	93.1%
FIB‐4 then FAP Index	94.5%	83.3%	18.0%	83.3%	94.5%	91.8%
NFS then FAP Index	91.8%	64.7%	25.8%	73.3%	88.2%	87.5%
FIB‐4 then NFS	94.2%	66.7%	21.3%	80.0%	89.1%	87.1%
NFS then FIB‐4	93.9%	57.1%	21.3%	80.0%	83.6%	82.9%

	Validation cohort					
	NPV	PPV	% Indeterminate	Sensitivity^a^	Specificity^a^	Accuracy
FAP Index	97.5%	53.8%	31.3%	58.4%	97.1%	94.9%
FIB‐4	95.7%	66.7%	24.1%	37.5%	98.7%	94.6%
NFS	93.2%	15.9%	47.5%	58.8%	64.4%	63.9%
FAP Index then FIB‐4	96.4%	55.6%	15.8%	52.6%	96.8%	93.6%
FAP Index then NFS	95.7%	29.3%	13.9%	54.5%	88.4%	85.7%
FIB‐4 then FAP Index	95.6%	53.3%	15.8%	42.1%	97.2%	93.2%
NFS then FAP Index	96.0%	15.9%	16.1%	55.6%	78.5%	76.5%
FIB‐4 then NFS	94.6%	29.7%	11.7%	45.8%	89.8%	86.0%
NFS then FIB‐4	94.3%	17.9%	11.7%	50.0%	78.4%	76.0%

^a^ Sensitivity and specificity were calculated with the indeterminate results excluded.

### 3.3 Comparisons Between FAP Index, NFS and FIB‐4

Comparisons of NITs were made in subcohorts (training *n* = 87, validation *n* = 316) that contained no missing data (Supplement S1.2, Supplementary Table 3). FAP Index accuracy was comparable to NFS and FIB4 (Table 3, Figure 1A; AUROC 0.842, 0.779, and 0.894, respectively).

Venn diagrams indicated classification overlaps and exclusivities when using FAP Index, FIB‐4 or NFS (Supplementary Figure 7). In the training subcohort, FAP Index correctly classified six F3–F4 patients to high risk that had not been so classified by the other NITs (Supplementary Figure 7A). Approximately 30% of patients were classified low risk by all three NITs, and ~70% of patients were classified low risk in two or more NITs, indicating overall agreement between NITs. NFS uniquely classified 29% of patients as indeterminate and classified far more as indeterminate than either FAP Index or FIB‐4, supporting superior accuracies of FAP Index and FIB‐4 to NFS. Moreover, more patients were classified high risk only by NFS (33%), and these patients were mostly F0–F2 misclassified as advanced fibrosis. More indeterminate classifications were uniquely made by FAP Index and FIB‐4 than NFS. Validation cohort Venn diagrams produced a similar, stronger pattern (Supplementary Figure 7B). Thus, these Venn diagrams depict both concordance between NITs, particularly FAP Index with FIB‐4, and potential FAP Index/FIB‐4 synergy.

FAP Index, FIB‐4, and NFS NPVs were 95.7%, 95.9%, and 92.9%, with specificities of 97.8%, 94%, and 81.2%, sensitivities of 80%–85%, and 36%, 31.5%, and 49.4% of patients classified as indeterminate, respectively, in the training cohort (Figure 2A,B, Supplementary Figure 6A, Table 3).

The validation cohort produced FAP Index and FIB‐4 specificities of ~98%, superior to NFS (64.4%). All NPVs were above 90% (Figure 2E–F, Supplementary Figure 6F, Table 3). Sensitivities for FAP Index and NFS were over 50%, but 37.5% for FIB‐4. FAP Index, FIB4, and NFS strongly correlated (*p* < 0.0001; Supplementary Figure 4B,D) and agreed in ~70% of patients, especially in the low‐risk classification (Supplementary Figure 7B). Importantly, all NITs exhibited large proportions of indeterminate (FAP Index 31.3%; FIB‐4 24.1%; NFS 47.5%), which suggested potential advantage in combining NITs.

### 3.4 Sequential Application of NITs

Each NIT generated classification exclusivities, so a method of two‐step sequential application of NITs was implemented (generic flowchart; Supplementary Figure 8). Each NITs combination reduced the indeterminate percentage while compromising neither diagnostic accuracy nor NPV (Table 3). Sequentially combining FAP Index with FIB‐4 or NFS reduced uncertainty by halving indeterminates to 14%–16% in the validation cohort and greatly reducing the proportion of indeterminate results to ~20% in the training cohort (Table 3, Figure 2C,D,G,H; Supplementary Figure 6B,C,G,H).

**FIGURE 3:**
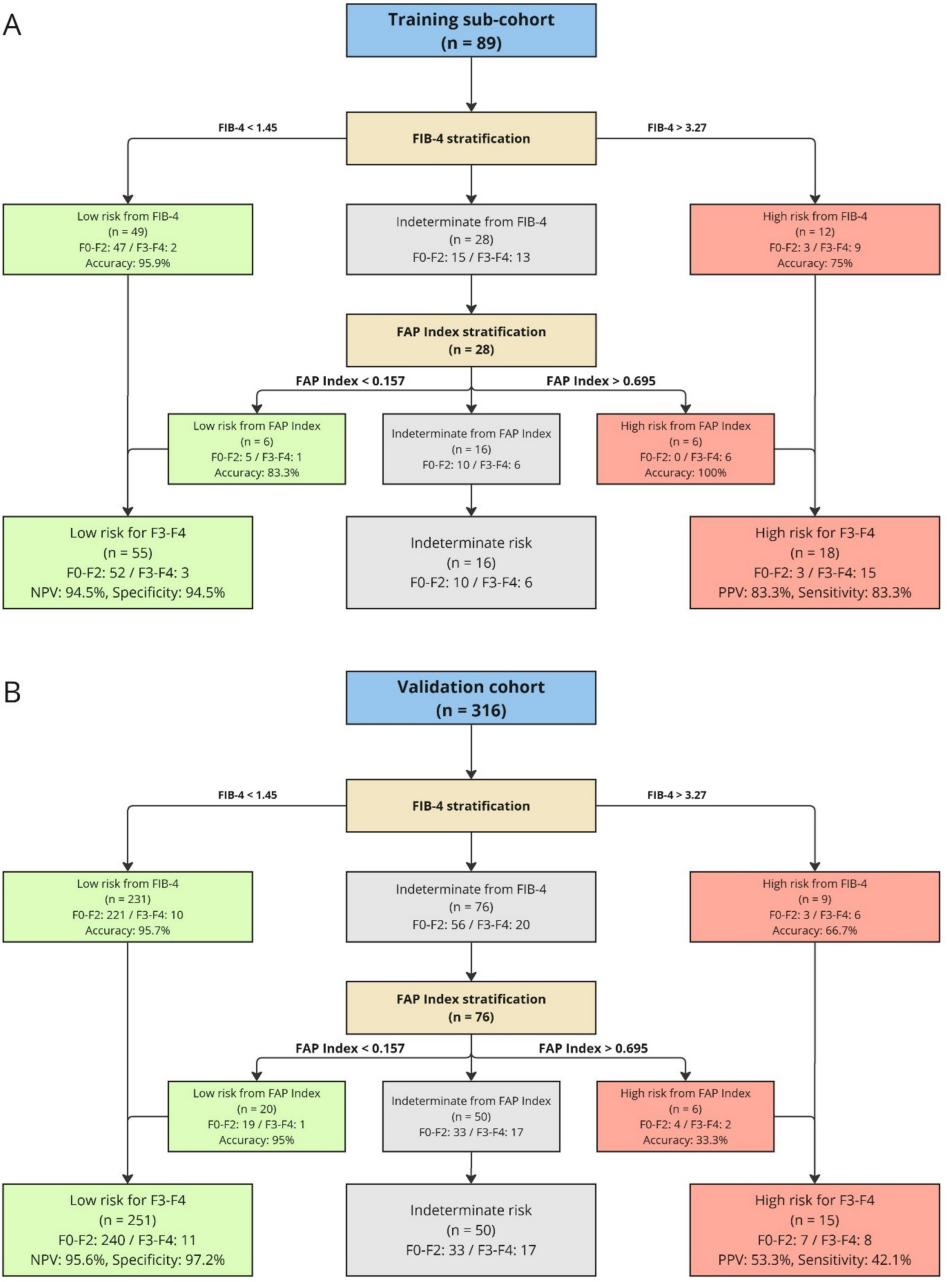
Flowchart for risk stratification using two sequential tests. FAP Index following FIB‐4 for discriminating the presence of advanced fibrosis in (A) training subcohort (*n* = 89) and (B) validation cohort (*n* = 316). FIB‐4 and FAP Index cutoffs for classification as indeterminate in each algorithm are shown.

FIB‐4 followed by FAP Index did not change sensitivity (training cohort 85%, validation cohort 42%) or PPV and retained excellent, ~95% specificity (Table 3). In contrast, combinations with NFS provided similar NPVs and rates of indeterminate sensitivity and specificity, but PPV fell to ~60% and < 30% in the two cohorts. In addition, comparing FIB‐4 then FAP Index (Table 3, Figure 3) with FIB‐4 then NFS (Supplementary Figure 9), the stepwise accuracy was greater when indeterminates were reclassified by FAP Index than by NFS. Including NFS in combinations produced less specificity and accuracy (Table 3, Figure 2D,H, Supplementary Figures 6), and, in the validation cohort, which had a lower prevalence of advanced fibrosis, NPV was greater and PPV smaller compared to the training subcohort. Stepwise flowcharts of all other combinations are presented (Supplementary Figures 10–13).

Overall, FIB‐4 followed by FAP Index generated superior diagnostic metrics, reducing indeterminate results while retaining excellent NPV and PPV compared to single NITs. Specifically, FIB‐4 followed by FAP Index reduced indeterminate results by 30%–40% in both cohorts.

### 3.5 A Comparison of cFAP With Type III Collagen Expression (PRO‐C3)

Strong correlations between cFAP and FAP Index with PRO‐C3 and the ADAPT algorithm were observed in a subcohort (W) with ~20% advanced fibrosis (Supplement S2.3; Supplementary Figures 14 and 15). The FAP Index generated similar NPV as APAPT but greater specificity. However, the FAP Index produced an indeterminate group, whereas ADAPT has a single cutoff, without indeterminates (Supplementary Table 4, subcohort W). In sequential combinations of FIB‐4 or NFS followed by ADAPT, no combination was superior or inferior to the others.

### 3.6 FAP Enzyme Assay Specificity and Reproducibility

The specificity of our assay for FAP [24] was reaffirmed in two independent laboratories (MDG and PVDV; Supplement S2.4, Supplementary Figure 16). Assay reproducibility with freeze/thaw, prolonged storage, assay timing, and change of personnel was shown (Supplement S2.5; Supplementary Figure 17), consistent with our previous reproducibility data [24, 26].

## 4 Discussion

In this study, the FAP Index was found to have a competitive discriminative ability to FIB‐4 for advanced liver fibrosis in MASLD populations. Most importantly, applying FAP Index following FIB‐4 produced far fewer indeterminate results without compromising diagnostic accuracy.

NITs are now commonly employed in early decision making in the care of patients with metabolic dysfunction and encouraged in current hepatology and diabetology guidelines [10, 13, 14]. Currently, FIB‐4 in combination with transient elastography is prominent in fibrosis screening. FAP Index following FIB‐4 could significantly assist triage in primary care settings and FAP Index requires only a FAP enzyme assay that is as simple as an ALT. Most fibrosis screening is at the community level, where transient elastography access is limited, especially outside cities, and our proposed FIB‐4/FAP Index strategy does not require dedicated hardware and is likely to reduce total diagnostic expenditures compared to elastography or expensive serum NITs.

FAP is an extracellular matrix and coagulation associated enzyme that is a marker of activated HSC and myofibroblasts and thus a direct fibrosis marker [15, 16, 17, 18, 19, 20]. Ordinal cFAP was incorporated into a novel NIT algorithm, FAP Index, and shown, when applied following or preceding NFS or FIB‐4, to greatly diminish the number of indeterminate outcomes without compromising sensitivity or specificity for advanced hepatic fibrosis. This improved diagnostic accuracy was both shown in a training cohort and validated in a pooled cohort of individuals with MASLD with differing prevalences of biopsy‐proven advanced fibrosis, type 2 diabetes, obesity, and metabolic syndrome. Measuring cFAP requires only one reagent (the fluorescent substrate) and a regular fluorescence reader, not a specialized machine. FAP Index uses cFAP, age, T2DM status, and ALT. The FIB‐4 algorithm similarly uses age and ALT but may be complementary to FAP Index because FIB‐4 uses platelet count.

Of the variables in FAP Index, ALT is a biomarker for epithelial cell injury, age is a known risk factor for liver disease progression [4], and T2DM and insulin resistance are drivers of MASLD progression [5]. Increases in these variables align with a dysmetabolic state with liver injury. FAP is strongly associated with the presence of advanced fibrosis [15, 21, 25], and activated HSC and myofibroblasts make and release FAP [15, 21, 39, 40] that contribute to collagen turnover [15, 17, 39]. Furthermore, recent FAP‐targeted radionuclide‐based 3D clinical imaging shows that intrahepatic FAP strongly aligns with fibrosis severity [22, 23]. Moreover, liver transplantation lowers cFAP [26], so the liver is a major origin of increased cFAP in patients with advanced fibrosis. The association between cFAP and HOMA2‐IR aligns with evidence that FAP is also a steatosis driver [36, 41]. Longitudinal studies could evaluate FAP Index in monitoring treatment outcomes.

Current NITs rely upon parameters associated with liver function, liver injury and inflammation, rather than directly with fibrosis [3, 8, 9, 11, 12]. Moreover, current NITs can yield large proportions of misclassifications [11, 12]. In contrast, FAP Index directly reflects the fibrotic process and shows strong discriminating capability among cohorts of varied patient composition and prevalence of advanced fibrosis. AUROCs were comparable between NITs, but FAP Index resolved many indeterminate results by sequentially applying FAP Index, probably because FAP Index reflects mesenchymal cell activation whereas FIB‐4 relates to platelets, so the two NITs are complementary. FAP Index was equally effective when either following or preceding either FIB‐4 or NFS: reducing indeterminate results by 30%–70% while not impairing NPV, PPV, specificity, or accuracy. Indeed, we and others have shown previously that sequential screening tests that employ dual cutoffs can increase the diagnostic accuracy of NITs and thereby reduce the number of biopsy referrals [11, 42, 43, 44]. Similarly, the current EASL algorithm recommends a stepwise strategy to increase diagnostic accuracy [8]. FIB‐4 is the most recommended NIT but produces ~35% indeterminate results in primary care cohorts [8]. In contrast, our two‐step strategy of FAP Index following FIB‐4 resulted in only 22% indeterminate when the advanced fibrosis prevalence was 11% (validation cohort), similar to the community [32, 33]. Increased specificity, with false positives and false negatives, was an additional advantage of sequentially combining FAP Index with FIB‐4. Therefore, FAP Index is potentially very beneficial for community screening.

The different prevalences of advanced fibrosis in the training and validation cohorts indicated that FAP Index may be useful in both primary and tertiary care settings. FAP Index was more accurate for ruling in advanced fibrosis in the tertiary‐like training cohort (20% fibrosis) than in the primary care‐mimicking validation cohort (11% fibrosis). In primary care, FAP Index may be useful as a longitudinal monitoring tool for changes in fibrosis severity. Furthermore, the high negative predictive value observed during validation underscores its potential utility in minimizing missed cases of advanced fibrosis.

FAP Index had comparable accuracy with another marker of activated fibroblasts, a precursor form of type III collagen, PRO‐C3, in a tertiary care subcohort. The PRO‐C3–based ADAPT algorithm has a single cutoff and thus no indeterminates [31], in this study, could only be a 2nd line test, following other NITs. Consistent with previous studies [45, 46, 47], ADAPT was superior to the three other NITs for ruling in advanced fibrosis in tertiary care. These outcomes are consistent with FAP Index, FIB‐4, and NFS suitability to primary care screening.

FAP Index can be a readily applied primary care test because it contains variables that are easily obtained, and cFAP measurement requires only a standard fluorescence measurement device, comparable with AST and ALT. Using ordinal cFAP in FAP Index confers potential applicability to a point‐of‐care lateral flow test. FAP Index as an accurate blood test and point‐of‐care test in routine screening potentially could alleviate total health care costs while fulfilling the increasing need for fibrosis testing. The enzyme activity of cFAP in serum is stable with cool storage and extended ultracold storage [24] (Supplement S2.2), which suits FAP Index as a reflex test following FIB‐4 within clinical pathology services.

FIB‐4 is most commonly recommended in standard of care guidance for liver fibrosis screening. However, current NITs rely upon parameters associated with liver function, liver injury, and inflammation, rather than directly with fibrosis [3, 8, 9, 11, 12]. Moreover, current NITs can yield large proportions of misclassifications [11, 12]. Liver fibrosis reflects tissue remodeling and myofibroblast activation. Therefore, including a simple biomarker such as FAP that directly reflects the fibrotic process is advantageous.

A major strength of this study was the robust training and validation cohorts. Fibrosis prevalence in the training cohort was similar to hepatology clinic populations, while the validation cohort mimicked community screening of at‐risk patients. Our simple one‐step assay to specifically quantify FAP enzyme activity showed excellent reliability and accuracy, without specialized hardware. We used validated methodology to devise the FAP Index and evaluate it. The study limitations were sample selection, sample size, and noncentralized histologic fibrosis assessment.

## 5 Conclusion

This study developed and validated FAP Index as a simple and novel NIT for triaging the risk of advanced fibrosis in MASLD. FAP Index demonstrated excellent diagnostic accuracy, with an AUROC of 0.875. In addition, sequential use of FAP Index following FIB‐4 produced few false negatives, with NPV > 95%, and produced ~30%–50% reductions in indeterminately classified patients compared to FIB‐4 alone. FAP Index includes a direct marker of fibrogenesis (cFAP) that is simple and inexpensive to measure and has potential applications as a point of care or reflex test. Thus, FAP Index demonstrates multiple advantages over the current first‐line standard of care NITs for triaging risk of advanced fibrosis in MASLD in primary care and should be further developed for clinical use.

## Funding

This work was supported by the National Health and Medical Research Council (APP1053206, APP1105238, APP1107178, APP1108422), Rebecca L. Cooper Medical Research Foundation, Centenary Institute Foundation (2021/ATRG2028), Gilead Fellowship, Robert W. Storr Bequest to the Sydney Medical Foundation, Hollywood Private Hospital Research Foundation, and Sir Charles Gairdner Osborne Parke Health Care Group.

## Ethics Statement

Ethics approval was obtained and approved separately by an independent ethics committee at each collection site. This study was conducted in accordance with the ethical principles enunciated in the World Medical Association Declaration of Helsinki.

## Consent

Written consent was obtained from all study participants prior to initiating any study procedures

## Conflicts of Interest

The authors declare no conflicts of interest.

## Supporting information


**Table S1:** jgh70294‐sup‐0001‐Supporting_Information.docx. **Patient characteristics for each individual cohort**. Statistically significant differences among all three cohorts (*p* value). Statistically significant differences between training cohort and Alfred Hospital cohort (cohort G [1]) or Westmead Hospital cohort (cohort W [2]) were obtained by post hoc (Tukey's) multiple comparison tests and are indicated by asterisks. Data presented as median ± IQR for continuous variables.
**Table S2:** Correlation analyses between circulating FAP activity (cFAP) and each other parameter in each cohort. Data presented as Pearson coefficient (*p* value). #: The value 1 was assigned to individuals with type 2 diabetes.
**Table S3:**. Baseline characteristics of the training cohort and the training subcohort. Mann–Whitney *U* test.
**Table S4:** Summary table for classification analyses of four NITs and NIT combinations in cohort W (*n* = 138), reported for NPV, PPV, proportion of indeterminate, sensitivity, specificity and accuracy. *Sensitivity and Specificity were calculated with the indeterminate results excluded.
**Figure S1: Ordinal cFAP.** (A) Histogram of cFAP activity in the training cohort. (B) Histogram of grouped/ordinal level of cFAP activity in the training cohort.
**Figure S2: Associations of cFAP activity and FIB‐4 with fibrosis staging**. The cFAP activity (U/L) (A, B) and FIB‐4 (C, D) segregated according to fibrosis stage are displayed for training cohort (*n* = 160) (A, C) and validation cohort (*n* = 332) (B, D, F). Dot plot with mean ± SEM. Differences between groups were determined using a one‐way ANOVA with Tukey's post hoc test. Significant differences are indicated with asterisks to indicate degree of difference: *p < 0.05, **p < 0.01, *** *p* < 0.001, **** *p* < 0.0001.
**Figure S3: Principal components analysis (PCA) of components of the FAP Index algorithm, showing individual data projected onto the first two principal components.** Assessment of the indeterminate interval in FAP Index when applied to the training cohort. PCA analysis of (A) low‐risk group compared with indeterminate outcomes that did not have advanced fibrosis and of (B) high‐risk group compared with indeterminate outcomes that had advanced fibrosis. The components of FAP Index were ordinal cFAP, age, type 2 diabetes status, and ALT. Each data point represents an individual observation, colored and shaped by risk group, with the mean of each group shown as an enlarged point. Each ellipse encompasses the 95% confidence interval for each group.
**Figure S4: Correlation analyses.** Linear correlation analyses of FAP Index with FIB‐4 (top) and with NAFLD Fibrosis Score (NFS; bottom) in the training **(A, C)** and validation **(B, D)** cohorts. Log10 transformation was applied to normalize skewed data points. Fibrosis stage was derived from scoring biopsies.
**Figure S5: ROC curves.** These ROC curves for established NITs illustrate the overall discriminative performance of these three NITs in the validation cohort. AUROC, area under curve; CI, confidence interval.
**Figure S6: Fibrosis classification accuracy.** Stratification for the risk of advanced fibrosis using the serum‐based NITs FAP Index, FIB4 and NFS, and sequential combinations of these NITs in the training (A–E) and validation (F–J) cohorts. Colors represent F0–F2 (green) and F3–F4 (red) biopsy‐derived fibrosis score.
**Figure S7:** Venn plots of classification of fibrosis risk as low, indeterminate or high by each algorithm applied to (A) training cohort and (B) validation cohort. Each percentage is % of total data in that Venn plot (*n*).
**Figure S8:** Diagnosis flowchart. Generic flowchart of a two‐step sequential application of noninvasive blood biomarker scoring classification.
**Figure S9: Diagnosis flowchart.** Flowchart for a risk stratification using two sequential NITs; NAFLD Fibrosis Score (NFS) following FIB‐4, to discriminate the presence of advanced fibrosis in (A) training subcohort (*n* = 89) and (B) validation cohort (*n* = 316). FIB‐4 and NFS cutoffs for indeterminate classification in each algorithm are shown.
**Figure S10: Diagnosis flowchart.** Flowchart for a risk stratification using two sequential NITs; FIB‐4 following NAFLD Fibrosis Score (NFS), to discriminate the presence of advanced fibrosis in (A) training subcohort (*n* = 89) and (B) validation cohort (*n* = 316). FIB‐4 and NFS cutoffs for indeterminate classification in each algorithm are shown.
**Figure S11: Diagnosis flowchart.** Flowchart for a risk stratification using two sequential NITs; FAP Index following NAFLD Fibrosis Score (NFS), to discriminate the presence of advanced fibrosis in (A) training subcohort (*n* = 89) and (B) validation cohort (*n* = 316). FIB‐4 and FAP Index cutoffs for indeterminate classification in each algorithm are shown.
**Figure S12: Diagnosis flowchart.** Flowchart for a risk stratification using two sequential NITs; FIB‐4 following FAP Index, to discriminate the presence of advanced fibrosis in (A) training subcohort (*n* = 89) and (B) validation cohort (*n* = 316). FIB‐4 and FAP Index cutoffs for indeterminate classification in each algorithm are shown.
**Figure S13: Diagnosis flowchart.** Flowchart for a risk stratification using two sequential NITs; NAFLD Fibrosis Score (NFS) following FAP Index, to discriminate the presence of advanced fibrosis in (A) training subcohort (*n* = 89) and (B) validation cohort (*n* = 316). FIB‐4 and FAP Index cutoffs for indeterminate classification in each algorithm are shown.
**Figure S14: Comparisons of cFAP based** FAP Index with PRO‐C3–based ADAPT. Evaluation of FAP‐related and PRO‐C3‐related variables for diagnostic potential in identifying advanced fibrosis in cohort W (A, B). Violin plots of (A) cFAP activity and (B) PRO‐C3 of patients without (No) and with (Yes) advanced fibrosis. Both cFAP activity and PRO‐C3 levels were significantly greater in advanced fibrosis. (C) Correlation matrix of cFAP Activity, FAP Index algorithm, PRO‐C3, and ADAPT algorithm, showing the Pearson correlation coefficients (*r*). (D, E) Scatter plots showing relationships between (D) log10‐transformed cFAP versus log10‐transformed PRO‐C3 levels, and (E) ADAPT with FAP Index, colored by fibrosis stage, with an ellipse drawn around 50% CI. (F) Receiver operating characteristic (ROC) for advanced fibrosis using FAP Index, ADAPT, FIB‐4, and NFS. (G–I) Stratification for risk of advanced fibrosis using FAP Index and ADAPT.
**Figure S15: Comparisons of ADAPT with other NITs.** Scatter plot showing the relationship between (A) log10‐transformed FAP Index and log10‐transformed ADAPT, (B) ADAPT algorithm versus NFS, colored by fibrosis stage, ellipse showing 50% CI. (C–L) Stratification for the risk of advanced fibrosis using serum‐based NITs in the training cohort, as an indicator of ***classification accuracy.*** F0–F2 (green) and F3–F4 (red) biopsy‐derived fibrosis score.
**Figure S16:** 3144‐AMC is a specific substrate of fibroblast activation protein alpha (FAP). An in‐house enzyme‐based assay was used to examine hydrolysis by (A) purified FAP, (C) purified DPP4 and (D) recombinant prolyl endopeptidase (PREP) in the presence of 3144‐AMC (blue), compound 4 (orange) and compound 6c (pink). Enzyme activity of (B) FAP (n = 5) and (E) PREP (n = 5) was calculated on the linear segment of each fluorescence plot. All three substrates were each used to determine hydrolysis by human serum (F) and thus enzyme activity (G) (n = 5). The three substrates were also used to measure substrate hydrolysis in the serum of wild‐type mice (H, I) (n = 4) and FAP enzyme negative (FAP−/−) mice (J) (n = 4), by published methods [3]. These compounds were provided by WWB (3144) and PVDV (compound 4 and compound 6c). Fluorescence was measured every 2.5 min for 1 h at 37°C in a plate reader with excitation at 355 nm and emission at 450 nm. Mean ± SEM.
**Figure S17:** Reproducibility of cFAP measurement by independent operators at different times. Pairwise analysis of cFAP activity measured at two different time points and performed by two different persons on serum samples from 150 patients of Westmead Hospital (cohort W). (A) The histogram of cFAP activity transformed into the ordinal levels 0, 1 and 2. The independent chi‐square test showed no significant difference in the distribution of the ordinal rank (χ2 = 0.1513, p = 0.93). (B) Paired t test showed no significant difference between the two measurements (t149 = 1.595, p = 0.11). (C) Paired t test showed no significant statistical difference between the two measurements of FAP Index calculated using the old and new cFAP activity measurements (t149 = 0.39, p = 0.70).

## Data Availability

The data that support the findings of this study are available on request from the corresponding author. The data are not publicly available due to privacy or ethical restrictions.
