# Supplementary material for: A Novel Fibroblast Activation Protein‐Based Algorithm to Assess Fibrosis in Metabolic Dysfunction–Associated Steatotic Liver Disease
Source: J Gastroenterol Hepatol. 2026 Feb 16;41(4):1311–22. doi: 10.1111/jgh.70294 (PMC13058791; doi:10.1111/jgh.70294)
Supplement: Supplementary file 1 — Table S1: jgh70294‐sup‐0001‐Supporting_Information.docx. Patient characteristics for each individual cohort. Statistically significant differences among all three cohorts (p value). Statistically significant differences between training cohort and Alfred Hospital cohort (cohort G [1]) or Westmead Hospital cohort (cohort W [2]) were obtained by post hoc (Tukey's) multiple comparison tests and are indicated by asterisks. Data presented as median ± IQR for continuous variables. Table S2: Correlation analyses between circulating FAP activity (cFAP) and each other parameter in each cohort. Data presented as Pearson coefficient (p value). #: The value 1 was assigned to individuals with type 2 diabetes. Table S3:. Baseline characteristics of the training cohort and the training subcohort. Mann–Whitney U test. Table S4: Summary table for classification analyses of four NITs and NIT combinations in cohort W (n = 138), reported for NPV, PPV, proportion of indeterminate, sensitivity, specificity and accuracy. *Sensitivity and Specificity were calculated with the indeterminate results excluded. Figure S1: Ordinal cFAP. (A) Histogram of cFAP activity in the training cohort. (B) Histogram of grouped/ordinal level of cFAP activity in the training cohort. Figure S2: Associations of cFAP activity and FIB‐4 with fibrosis staging. The cFAP activity (U/L) (A, B) and FIB‐4 (C, D) segregated according to fibrosis stage are displayed for training cohort (n = 160) (A, C) and validation cohort (n = 332) (B, D, F). Dot plot with mean ± SEM. Differences between groups were determined using a one‐way ANOVA with Tukey's post hoc test. Significant differences are indicated with asterisks to indicate degree of difference: *p < 0.05, **p < 0.01, *** p < 0.001, **** p < 0.0001. Figure S3: Principal components analysis (PCA) of components of the FAP Index algorithm, showing individual data projected onto the first two principal components. Assessment of the indeterminate interval in FAP Index when a [file JGH-41-1311-s001.docx]

Supplementary information for:

A novel fibroblast activation protein-based algorithm to assess fibrosis in metabolic dysfunction-associated steatotic liver disease

**Running title**: Novel blood test for liver fibrosis in MASLD

**Authors:** Ziqi V Wang BSc, Badwi B Boumelhem PhD, Torsten Pennell BSc, William W Bachovchin PhD, Jack Hung-Sen Lai PhD, Sarah E Poplawski PhD, Harsha Chandraratna MBBS, Pieter Van Der Veken PhD, Kate Brewer BSc, Diana Julie Leeming PhD, Geraldine Ooi MBBS, PhD, Jacob George MBBS, PhD, Mohammed Eslam MBBS, PhD, Leon A Adams MBBS, PhD, Hui Emma Zhang PhD, Geoffrey W McCaughan MBBS, PhD, Avik Majumdar MBBS, PhD* & Mark D Gorrell PhD*

Table of Contents

[S1 Supplementary Methods 2](#_Toc219831251)

[S1.1 | Study cohorts 2](#_Toc219831252)

[S1.2 | Clinical, demographic and histologic data of study cohorts 2](#_Toc219831253)

[S1.3 | The specificity of 3144-AMC for FAP 2](#_Toc219831254)

[S2 Supplementary Results 3](#_Toc219831255)

[S2.1 | Evaluation of thresholds for indeterminate classification in FAP-Index 3](#_Toc219831256)

[S2.2 | Correlation of FAP-Index with established NITs 3](#_Toc219831257)

[S2.3 | A comparison of FAP based with type III pro-collagen based variables 4](#_Toc219831258)

[S2.4 | Enzyme assay specificity for FAP 4](#_Toc219831259)

[S2.5 | Enzyme stability and FAP assay reproducibility 5](#_Toc219831260)

[Supplementary Tables 7](#_Toc219831261)

[Supplementary Figures 11](#_Toc219831262)

[References for Supplementary Information 30](#_Toc219831263)

**S1 Supplementary Methods**

## S1.1 | Study cohorts

The training cohort (n=160) consisted of 65 MASLD patients and 95 bariatric surgery patients from a hepatology clinic of Sir Charles Gairdner Hospital, Perth, Australia, and much of that cohort has been described [[1](#_ENREF_1)]. The validation cohort (n=332) was derived from two previously described study populations; 182 bariatric surgery patients of The Alfred Hospital, Melbourne, Australia [[2](#_ENREF_2)] and 150 MASLD patients of Westmead Hospital, Sydney, Australia [[3](#_ENREF_3)] Ethics approvals were HREC_X18-0241, HREC_X25-0176 and HREC_X19-0488 in Sydney Local Health District, 2019/ETH02319 in Westmead Hospital, RGS 01287 in Sir Charles Gairdner Hospital, and 195/15 in The Alfred Hospital.

## S1.2 | Clinical, demographic and histologic data of study cohorts

All data were de-identified. Demographic data and routine clinical laboratory values were obtained within four weeks of liver biopsy and measured in standardized units used in Australia. Histological fibrosis assessments, using the Kleiner scoring system, were provided by each hospital pathology service [[1-3](#_ENREF_1)]. The outcome variable of advanced liver fibrosis was defined as fibrosis staging > 2 (F3-F4). For 138 of the MASLD patients of Westmead Hospital, NordicPRO-C3 ELISA data and its processing in the ADAPT algorithm has been reported previously. HOMA2-IR was calculated using the HOMA2 model published by Oxford University Innovation [[7](#_ENREF_7)].

When comparing NITs using the training cohort, a large proportion of cases lacked data for all three NITs, so were excluded and thus the training sub-cohort contained fewer cases (n=87) than the training cohort (n = 160). Therefore, the training sub-cohort and training cohort were compared for statistical differences. Compared with the training cohort, the training sub-cohort was older (57 vs 52), had lower BMI (34 vs 38) and had higher insulin (15 vs 11) and cholestasis associated enzymes, but advanced fibrosis prevalence did not differ (Supplementary table 3).

## S1.3 | The specificity of 3144-AMC for FAP

The FAP-specific substrate, 3144-AMC (previously called ARI-3144-AMC) [[8](#_ENREF_8)], was compared to two other substrates; compound 4 (intended to be identical to 3144-AMC) [[9](#_ENREF_9)] and compound 6c [[9](#_ENREF_9)]. Each compound was solubilised in DMSO then diluted to a stock concentration of 10 mM, aliquoted and stored at -20 °C until diluted in Tris (10 mM) / EDTA (1 mM) pH 7.4 (TE buffer) for a final concentration of 150 µM in 100 µL, with dithiothreitol at 1 mM when detecting prolyl endopeptidase (PREP). Substrate hydrolysis by 0-10 ng purified recombinant DPP4 [[10](#_ENREF_10), [11](#_ENREF_11)], FAP [[10](#_ENREF_10), [11](#_ENREF_11)] and PREP (R&D Systems; 4308-SE-010) was measured using the DPP4 substrate H-Gly-Pro-pNA (Bachem, Bubenhof, Switzerland; catalog number 4025614) at 1 mM and the PREP substrate Z-Gly-Pro-pNA (Bachem catalog number 4003649) at 1 mM.

# S2 Supplementary Results

## S2.1 | Evaluation of thresholds for indeterminate classification in FAP-Index

Following FAP-Index optimisation, dual cutoffs produced sizeable indeterminate populations (Figure 1B) that contained many patients without advanced fibrosis.

To understand indeterminate populations, principal component analysis (PCA) indicated that amongst indeterminate patients without advanced fibrosis, some exhibited high cFAP and ALT and some exhibited T2DM and greater age (Supplementary figure 3A). In contrast, patients with an indeterminate score from FAP-Index who had advanced fibrosis were found to be spread along the principal component in which cFAP had a dominant contribution, whereas effects from ALT, T2DM and age were small (Supplementary figure 3B).

These analyses suggest that T2DM and age were overweighted as predictive variables, and that the strength of cFAP as a risk in the model was under-estimated.

## S2.2 | Correlation of FAP-Index with established NITs

FAP-Index correlations with currently established NITs were measured. FAP-Index exhibited a linear and positive regression with both FIB-4 and NFS across both training and validation cohorts (Supplementary figure 4). In the training cohort, the goodness-of-test analysis showed that FAP-Index moderately correlated with both FIB-4 Index (r2 = 0.25) and NFS (r2 = 0.23). In the validation cohort, FAP-Index correlated with FIB-4 Index (r2 = 0.41, r2 = 0.22, respectively), and with NFS (r2 = 0.2, r2 = 0.04, respectively).

## S2.3 | A comparison of FAP based with type III pro-collagen based variables

NordicPRO-C3 ELISA data has been published previously [[3](#_ENREF_3)] for one sub-cohort, a tertiary care liver clinic cohort, Cohort W (n=138). PRO-C3 data is central to the ADAPT algorithm, and ADAPT data was also available from DJL [[3](#_ENREF_3)]. Overall, the ALT and AST levels in cohort W were greater than in the training cohort.

Both cFAP (P = 0.014, Supplementary figure 14A) and PRO-C3 (P < 0.001, Supplementary figure 14B) were elevated with advanced fibrosis. The variables cFAP and PRO-C3 were positively correlated and explained 12% of the linear variance by R^2^ (Supplementary figure 14C-D). FAP-Index and ADAPT were strongly correlated, with 40% of variance explained (Pearson coefficient = 0.63; Supplementary figure 14C, E). FIB-4 and NFS were moderately correlated with ADAPT, with 35% of variance explained (Supplementary figure 15A-B).

Comparing accuracy of risk stratification against presence of advanced fibrosis, ADAPT performed best among the four NITs (AUROCs: ADAPT 0.854; FAP-Index 0.752, FIB-4 0.768, NFS 0.76). FAP-Index and ADAPT NPVs were 94% and 96% and PPVs 64% and 51%, respectively (Supplementary figure 14G-H). However, FAP-Index produced 45% (62/138) indeterminate in cohort W, mainly F0-F2. ADAPT has a single cutoff, 6.33, so had no indeterminates. Therefore, to evaluate sequential NITs, ADAPT can be a 2nd line but not a 1st line NIT. Sequential application of FAP-Index and ADAPT produced NPV 94% and PPV 53%, comparable with ADAPT alone (Supplementary figure 14I). Other NIT combinations were comparable (Supplementary figure 15C-L; supplementary table 4).

## S2.4 | Enzyme assay specificity for FAP

We previously demonstrated the specificity of 3144-AMC as a substrate for tissue derived mouse FAP, and for circulating fibroblast activation protein alpha (cFAP) in human and mouse plasma and serum [[8](#_ENREF_8)]. Furthermore, we discovered a ~20x increase in FAP activity in human cirrhotic liver compared to healthy liver [[12](#_ENREF_12)] and up to 20-fold more cFAP in mouse than in healthy human sera. Here, we have optimised the FAP enzyme activity assay and further showed the specificity of 3144-AMC for FAP and confirmed that compound 6c is also a specific substrate for FAP [[9](#_ENREF_9)] (Supplementary figure 2). 3144-AMC, compound 4 [[9](#_ENREF_9)] and compound 6c exhibited similar hydrolysis by FAP (Supplementary figure 2 A, B). Purified DPP4 did not detectably hydrolyse any of the three substrates (Supplementary figure 2C). Compound 4 was hydrolysed by recombinant PREP, whereas no hydrolysis was observed with either 3144-AMC or compound 6c (Supplementary figure 2 D, E). In the presence of human serum, all substrates exhibited similar FAP activity (Supplementary figure 2 F, G).

Finally, we assessed substrate hydrolysis by natural FAP that is in the serum of wild-type and FAP enzyme negative mice (Supplementary figure 2H-J). FAP activity measurements in the serum of wild-type mice were similar across all three substrates (Supplementary figure 2H-I). Most convincing of the specificity of 3144-AMC for FAP was the lack of FAP activity detected in the serum of FAP enzyme negative mice (Supplementary figure 2J), concurring with our previously published data [[8](#_ENREF_8)]. Taken together, these data clarify that compound 6c and 3144-AMC have equal potency and specificity for measuring FAP activity. The chemical structures of compound 6c and 3144-AMC are identical except for two fluorine substituents on the proline ring in compound 6c. Therefore, the above data shows that this structural difference is inconsequential for their interaction with FAP.

Compound 4 was synthesized to lack those fluorines and thus be identical to 3144-AMC [[9](#_ENREF_9)]. Why compound 4 was not as specific as 3144-AMC here or in the initial publication of compound 4 is unclear but may have been caused by a degree of stereochemical impurity at the Ala position. Also confirming the FAP specificity of 3144, newly synthesised compound 4 (3144), synthesised by an author of both the present and previous publications (PVDV) was specific for FAP over PREP and DPP4.

## S2.5 | Enzyme stability and FAP assay reproducibility

In this study, FAP stability and the reproducibility of the cFAP enzyme assay on human serum samples was examined by repeat assay of the entire Westmead Hospital cohort (cohort W) five years after cFAP was first measured for the initial data collection. No statistical difference was observed between these two replicate assays (P=0.11) (Supplementary figure 3). By transforming the cFAP activity into ordinal levels using the cut-offs mentioned above, the histograms for these two replicate assays were almost identical (Supplementary figure 3). Moreover, when each cFAP ordinal dataset from the assays performed five years apart by different personnel using different batches of reagents were incorporated into the FAP-Index calculation, no significant difference was identified (P=0.70). This was consistent with our previous finding that freeze/thaw cycles do not affect cFAP activity [[8](#_ENREF_8)], and strongly indicates the stability of cFAP activity in storage and a robust and very reproducible assay.

# Supplementary Tables

***Supplementary table 1.*** ***Patient characteristics for each individual cohort****. Statistically significant differences among all three cohorts (P-value). Statistically significant differences between training cohort and Alfred Hospital cohort (cohort G[*[*2*](#_ENREF_2)*]) or Westmead Hospital cohort (cohort W[*[*3*](#_ENREF_3)*]) were obtained by post hoc (Tukey’s) multiple comparison tests and are indicated by asterisks. Data presented as median ± IQR for continuous variables.*

|  | **Training cohort (n=160)** | **Validation cohort (n =332)** | | |
| --- | --- | --- | --- | --- |
|  | **Training cohort (n=160)** | **Alfred cohort (G)**  **(n=182)** | **Westmead cohort (W)**  **(n = 150)** | ***P-value*** |
| Age ^a^ | 52±18.25 | 45±19.75^****^ | 52±16 | <0.0001 |
| Gender (Male) ^b^ | 58 (36%) | 44 (24%) | 76 (50.7%) | <0.0001 |
| T2DM (1) ^b#^ | 57 (36%) | 41 (23%) | 57 (38%) | 0.004 |
| BMI ^a^ | 38.19±12.05 | 45.15±10.86^****^ | 30.82±7.48^****^ | <0.0001 |
| ALT (U/L) ^a^ | 40±42.25 | 33±26^*^ | 66.5±51^**^ | <0.0001 |
| AST (U/L) ^a^ | 32.5±20 | 27±13^*^ | 55.58±29^****^ | <0.0001 |
| GGT (U/L) ^a^ | 40±60.25 | 33±21^****^ | 85.5±94.5 | <0.0001 |
| PLT (x10^9^/L) ^a^ | 225±94 | 238.5±88.25 | 241±88.7 | 0.13 |
| ALP (U/L) ^a^ | 81±35.5 | 69±27 | Nd | Nd |
| Insulin (mU/L) ^a^ | 11±15 | 7.1±7.8^**^ | 15±12 | 0.002 |
| HOMA2-IR | 1.48 (2.07) | 0.94 (1.02) ^***^ | 1.96 (1.53) | <0.001 |
| Fibrosis staging ^b^ |  |  |  | <0.0001 |
| F0 | 81 | 139 | 41 |  |
| F1 | 35 | 36 | 48 |  |
| F2 | 10 | 3 | 27 |  |
| F3 | 17 | 3 | 26 |  |
| F4 | 12 | 1 | 8 |  |
| Advanced fibrosis ^b^ | 32 (20%) | 4 (2%) | 34 (22.7%) | <0.0001 |
| cFAP activity  (pmol AMC/min/L) ^a^ | 995.74±579.3 | 1193.22±434.04^**^ | 1419±635.1^****^ | <0.0001 |
| Ordinal cFAP ^b^ |  |  |  | <0.0001 |
| Level 0 | 38 | 11 | 4 |  |
| Level 1 | 94 | 142 | 87 |  |
| Level 2 | 28 | 29 | 59 |  |

* p-value<0.05, ** p-value<0.01, *** p-value<0.001, **** p-value<0.0001 of Mann-Whitney t-test comparison with cohort P.

^a^ One-way ANOVA was used to evaluate statistical significance of differences among means across all cohorts.

^b^ Chi-square test was used to identify statistically significant differences among proportions across all cohorts.

#: The value 1 was assigned to individuals with type 2 diabetes (T2DM).

***Supplementary table 2.*** *Correlation analyses between circulating FAP activity (cFAP) and each other parameter in each cohort. Data presented as Pearson coefficient (P-value). #: The value 1 was assigned to individuals with type 2 diabetes.*

|  | ***Training cohort*** | ***Validation cohort*** |
| --- | --- | --- |
| Age | 0.13 (0.11) | 0.12 (0.03) |
| Gender (Male) | 2050 (0.001) | 9337 (0.01) |
| T2DM ^#^ (1) | 3396 (0.10) | 11157 (0.74) |
| Hypertension | 3608 (0.14) | 13104 (0.56) |
| Weight (kg) | -0.04 (0.59) | 0.02 (0.82) |
| Height (m) | 0.10 (0.22) | 0.19 (0.009) |
| BMI | -0.11 (0.15) | -0.25 (<0.001) |
| Bilirubin (umol/L) | 0.11 (0.15) | 0.13 (0.08) |
| ALP (U/L) | 0.24 (0.002) | 0.13 (0.08) |
| ALT (U/L) | 0.28 (<0.001) | 0.19 (<0.001) |
| AST (U/L) | 0.36 (<0.001) | 0.18 (0.001) |
| GGT (U/L) | 0.34 (<0.001) | 0.13 (0.02) |
| AST/ALT | 0.02 (0.83) | 0.02 (0.7) |
| Alb (g/dL) | -0.14 (0.08) | 0.17 (0.001) |
| PLT (x10^9^/L) | -0.28 (0.004) | -0.06 (0.29) |
| Creatinine (umol/L) | 0.10 (0.35) | - |
| Glucose (mmol/L) | 0.004 (0.95) | -0.09 (0.26) |
| Insulin (mU/L) | 0.31 (<0.001) | 0.18 (0.001) |
| HOMA2-IR | 0.32 (<0.001) | 0.23 (<0.0001) |
| TG (mmol/L) | 0.04 (0.66) | 0.16 (0.003) |
| Steatosis | 5.96 (0.11) | 7.86 (0.049) |
| Inflammation | 3.45 (0.18) | 7.54 (0.05) |
| Ballooning | 9.10 (0.01) | 9.12 (0.01) |
| Fibrosis | 26.95 (<0.001) | 37.52 (<0.001) |

***Supplementary table 3****. Baseline characteristics of the training cohort and the training subcohort. Mann-Whitney U-test.*

|  | ***Training cohort (n=160)*** | ***Training sub-cohort (n=87)*** | ***P-value*** |
| --- | --- | --- | --- |
| Age | 52±18.25 | 57±15 | 0.03 |
| Gender (Male) | 58 (36%) | 34 (39.1%) | 0.76 |
| Diabetes (1) ^#^ | 57 (36%) | 34 (39.1%) | 0.69 |
| BMI | 38.19±12.05 | 34±11.4 | 0.02 |
| ALT (U/L) | 40±42.25 | 51±45.5 | 0.22 |
| AST (U/L) | 32.5±20 | 37±26.5 | 0.15 |
| GGT (U/L) | 40±60.25 | 61±97.5 | 0.02 |
| Platelets (PLT; x10^9^/L) | 225±94 | 216±101.5 | 0.57 |
| ALP (U/L) | 81±35.5 | 92±51 | 0.04 |
| Insulin (mU/L) | 11±15 | 15±17 | 0.02 |
| HOMA2-IR | 1.48 (2.07) | 2 (2.42) | 0.04 |
| Fibrosis staging |  |  | 0.68 |
| F0 | 81 (51.3%) | 38 (43.7%) |  |
| F1 | 35 (22.2%) | 19 (21.8%) |  |
| F2 | 10 (6.3%) | 6 (6.9%) |  |
| F3 | 17 (10.8%) | 11 (12.6%) |  |
| F4 | 15 (9.5%) | 13 (14.9%) |  |
| Advanced fibrosis | 32 (20.3%) | 24 (27.6%) | 0.25 |
| cFAP activity (pmol AMC/min/L) | 995.74±579.3 | 1075±616.5 | 0.56 |
| cFAP activity ordinal |  |  | 0.54 |
| Level 0 | 38 (23.8%) | 21 (24.1%) |  |
| Level 1 | 94 (58.5%) | 46 (52.9%) |  |
| Level 2 | 28 (17.5%) | 20 (23%) |  |

Notes:

p-value <0.05 indicates statistical significance.

Data presented as median ± IQR for continuous variables, and prevalence (%) for categorical variables.

#The value 1 was assigned to individuals with type 2 diabetes.

***Supplementary table 4.*** *Summary table for classification analyses of four NITs and NIT combinations in cohort W (n = 138), reported for NPV, PPV, proportion of indeterminate, sensitivity, specificity and accuracy. *Sensitivity and Specificity were calculated with the indeterminate results excluded.*

| **Test performed** | **Diagnostic matrices** | | | | | |
| --- | --- | --- | --- | --- | --- | --- |
|  | **Cohort W** | | | | | |
|  | **NPV** | **PPV** | **% Indeterminate** | **Sensitivity*** | **Specificity*** | **Accuracy** |
| FAP-Index | 93.8% | 63.6% | 44.9% | 63.6% | 93.8% | 89.5% |
| FIB-4 | 89.4% | 75.0% | 32.6% | 40.0% | 97.4% | 88.2% |
| NFS | 92.0% | 50.0% | 37.0% | 50.0% | 92.0% | 86.2% |
| ADAPT | 96.4% | 50.9% | 0.0% | 90.3% | 74.8% | 78.3% |
| FAP-Index then FIB-4 | 91.2% | 62.5% | 22.5% | 55.6% | 93.3% | 86.9% |
| FAP-Index then NFS | 90.9% | 50.0% | 23.2% | 52.9% | 89.9% | 84.0% |
| FAP-Index then ADAPT | 94.4% | 53.1% | 0.0% | 83.9% | 78.5% | 79.7% |
| FIB-4 then FAP-Index | 89.4% | 61.5% | 22.5% | 44.4% | 94.4% | 86.0% |
| NFS then FAP-Index | 92.0% | 55.6% | 23.2% | 58.8% | 91.0% | 85.8% |
| FIB-4 then NFS | 88.4% | 53.3% | 20.3% | 42.1% | 92.3% | 83.6% |
| NFS then FIB-4 | 88.4% | 53.3% | 20.3% | 42.1% | 92.3% | 83.6% |
| FIB-4 then ADAPT | 90.8% | 55.0% | 0.0% | 71.0% | 83.2% | 80.4% |
| NFS then ADAPT | 92.5% | 53.3% | 0.0% | 77.4% | 80.4% | 79.7% |

# Supplementary Figures


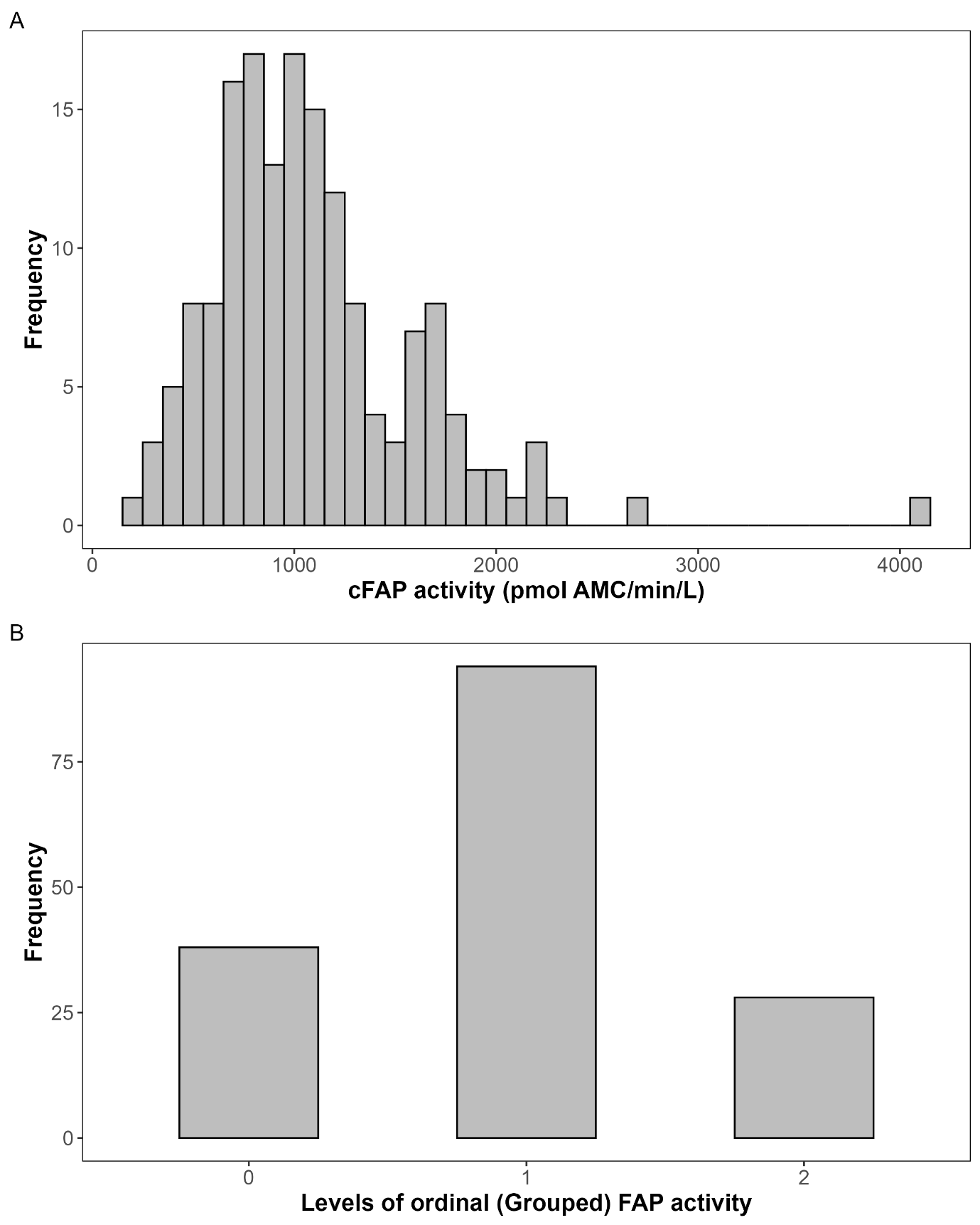


***Supplementary figure 1. Ordinal cFAP.*** A: Histogram of cFAP activity in the training cohort. B: Histogram of grouped/ordinal level of cFAP activity in the training cohort.


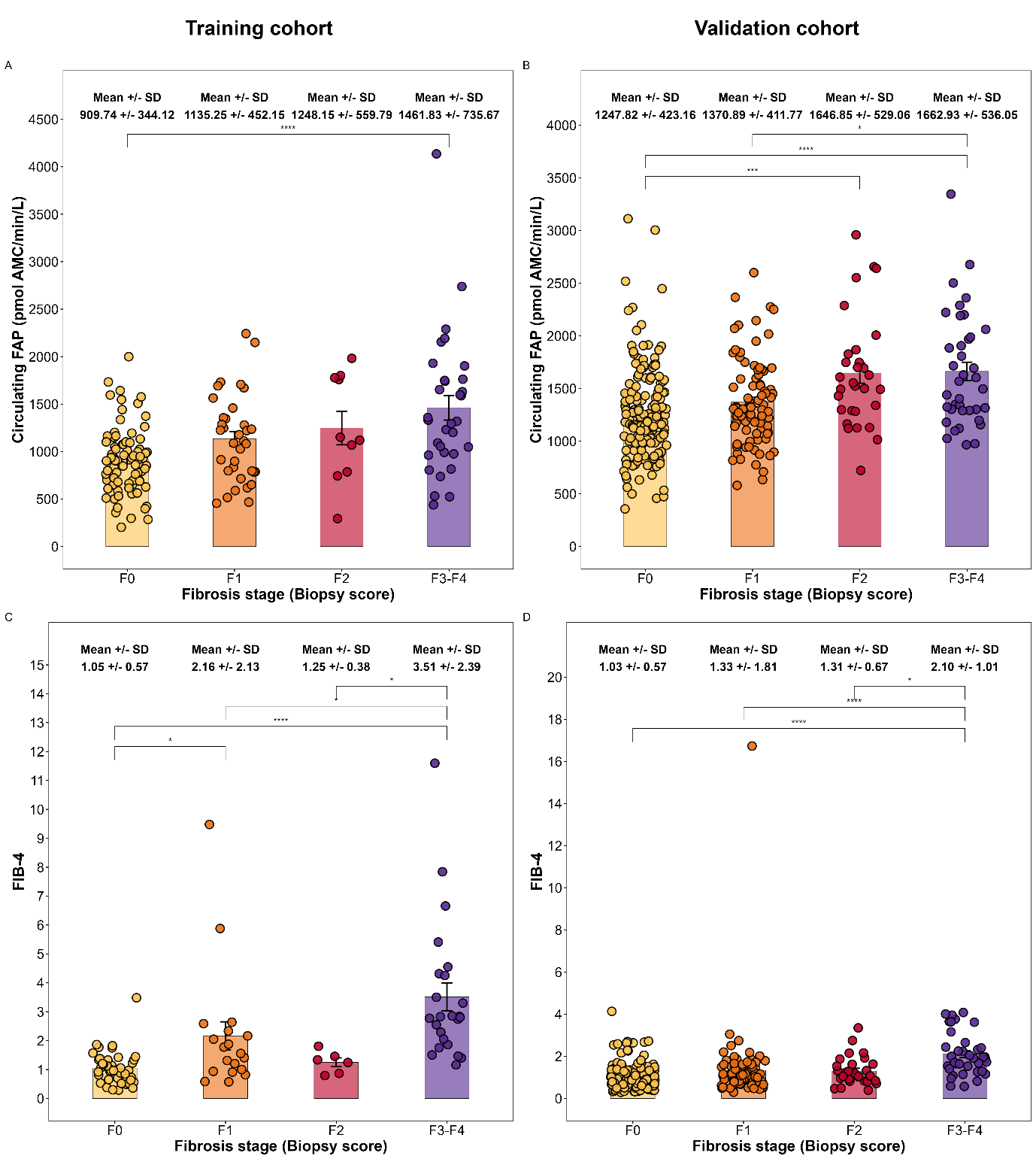


***Supplementary figure 2.*** ***Associations of cFAP activity and FIB-4 with fibrosis staging***. The cFAP activity (U/L) (A, B) and FIB-4 (C, D) segregated according to fibrosis stage are displayed for training cohort (n=160) (A, C) and validation cohort (n = 332) (B, D, F). Dot plot with mean ± SEM. Differences between groups were determined using a one-way ANOVA with Tukey’s post-hoc test. Significant differences are indicated with asterisks to indicate degree of difference: * p-value<0.05, ** p-value<0.01, *** p-value<0.001, **** p-value<0.0001.


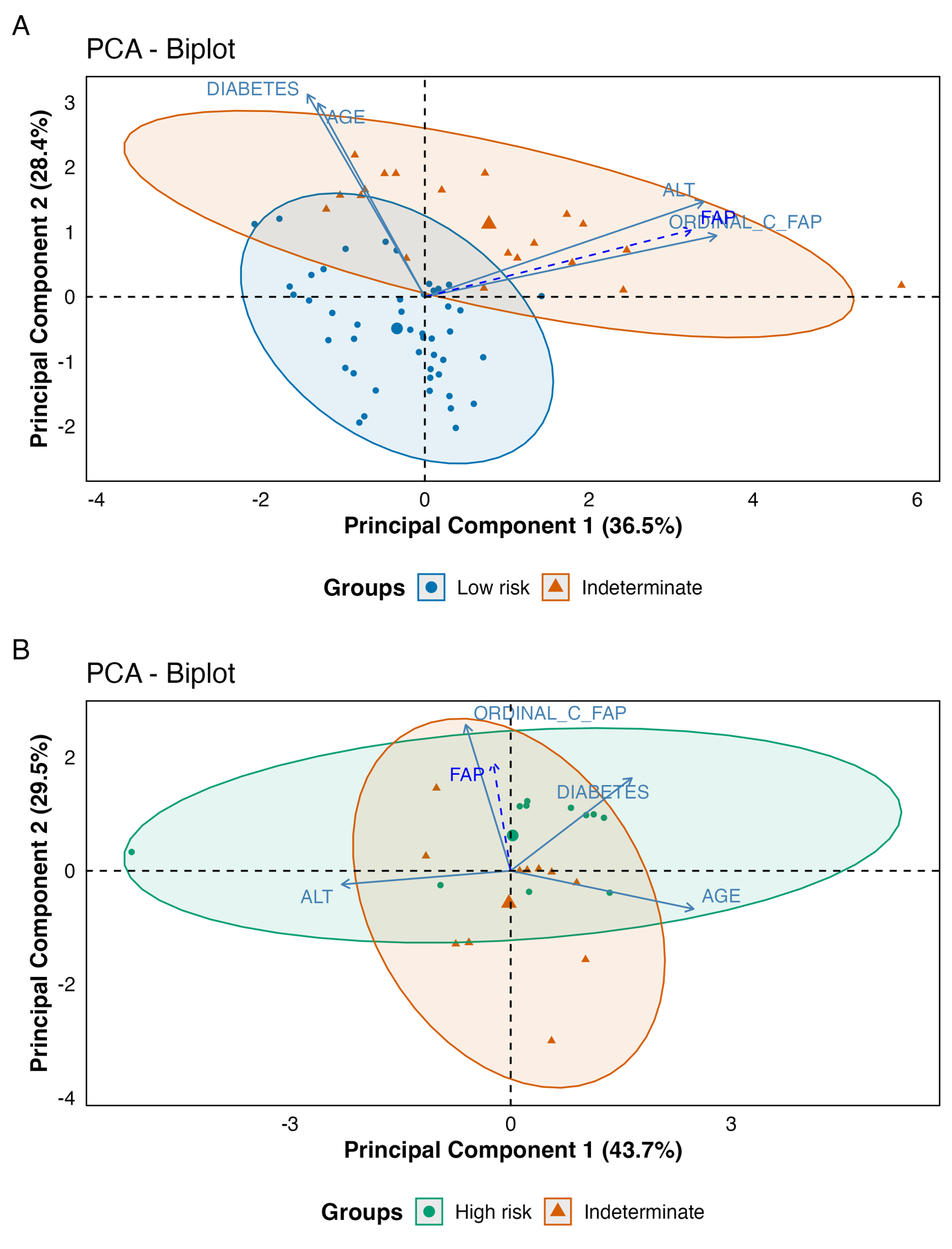


***Supplementary figure 3. Principal components analysis (PCA) of components of the FAP-Index algorithm, showing individual data projected onto the first two principal components.*** Assessment of the indeterminate interval in FAP-Index when applied to the training cohort. PCA analysis of (A) low-risk group compared with indeterminate outcomes that did not have advanced fibrosis, and of (B) high-risk group compared with indeterminate outcomes that had advanced fibrosis. The components of FAP-Index were ordinal cFAP, age, type 2 diabetes status and ALT. Each data point represents an individual observation, coloured and shaped by risk group, with the mean of each group shown as an enlarged point. Each ellipse encompasses the 95% confidence interval for each group.


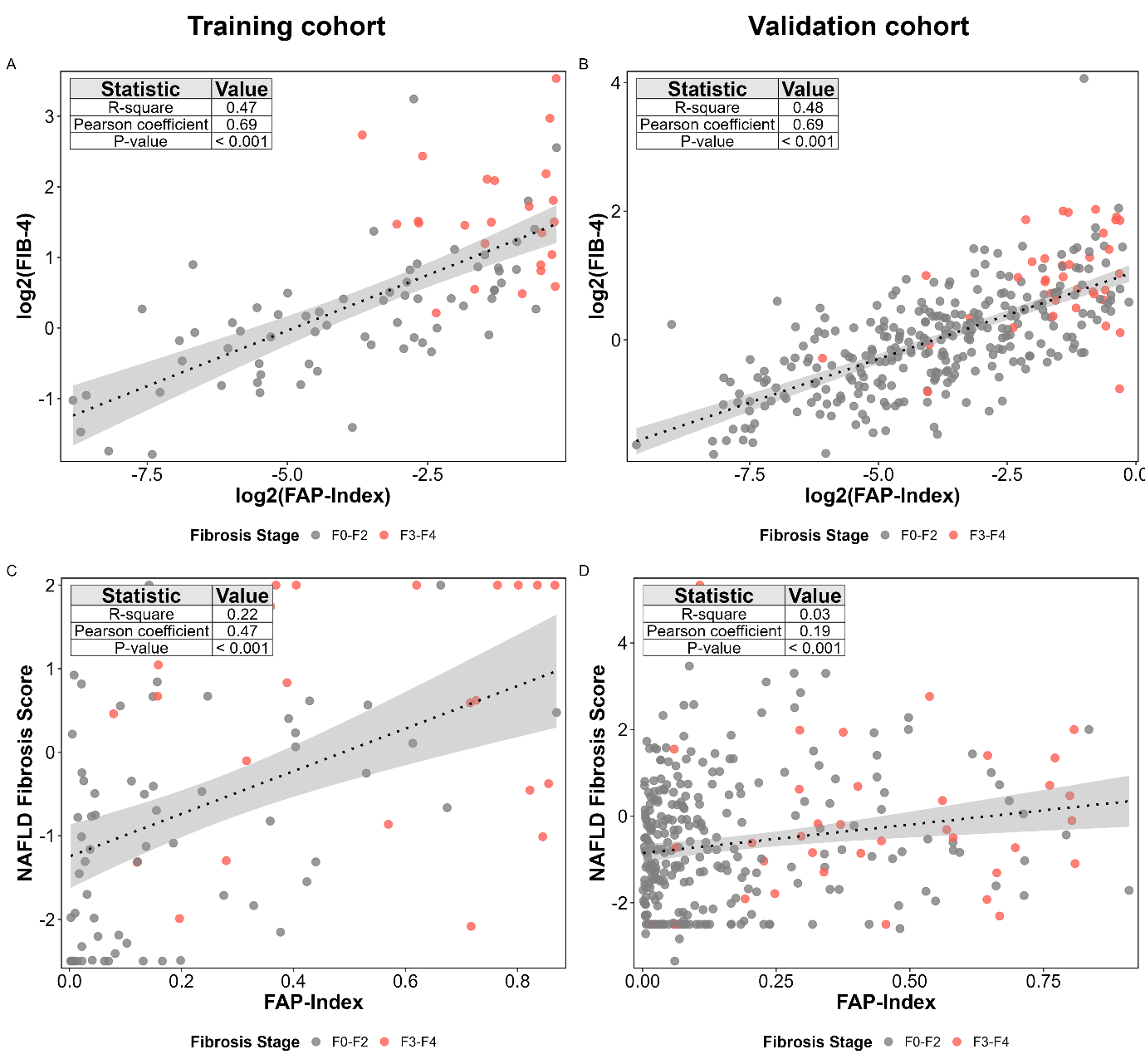


***Supplementary figure 4. Correlation analyses*.** Linear correlation analyses of FAP-Index with FIB-4 (top) and with NAFLD Fibrosis Score (NFS; bottom) in the training **(A, C)** and validation **(B, D)** cohorts. Log10-transformation was applied to normalize skewed data points. Fibrosis stage was derived from scoring biopsies.


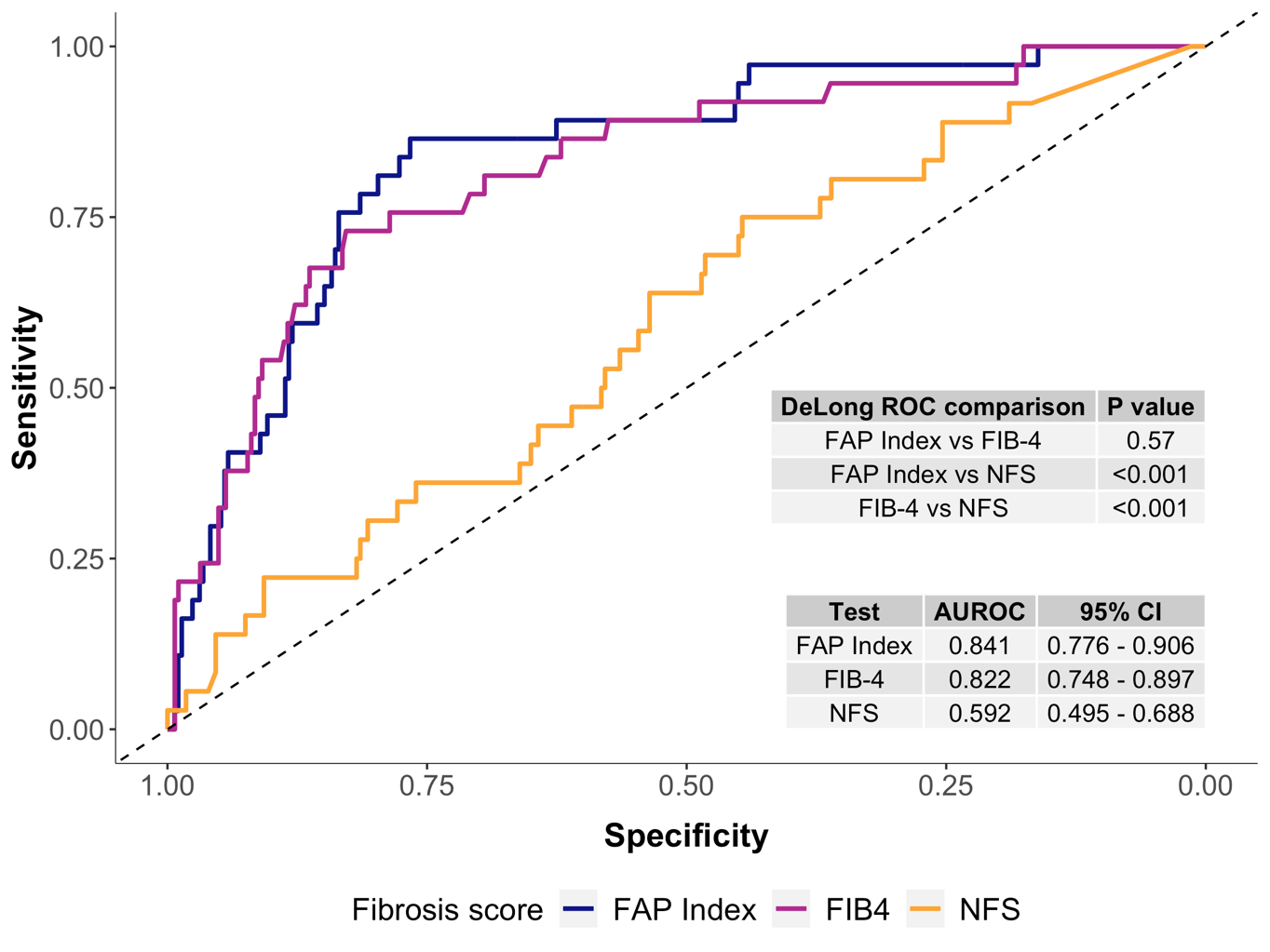


***Supplementary figure 5.*** ***ROC curves*.** These ROC curves for established NITs illustrate the overall discriminative performance of these three NITs in the validation cohort. CI: confidence interval. AUROC: area under curve.


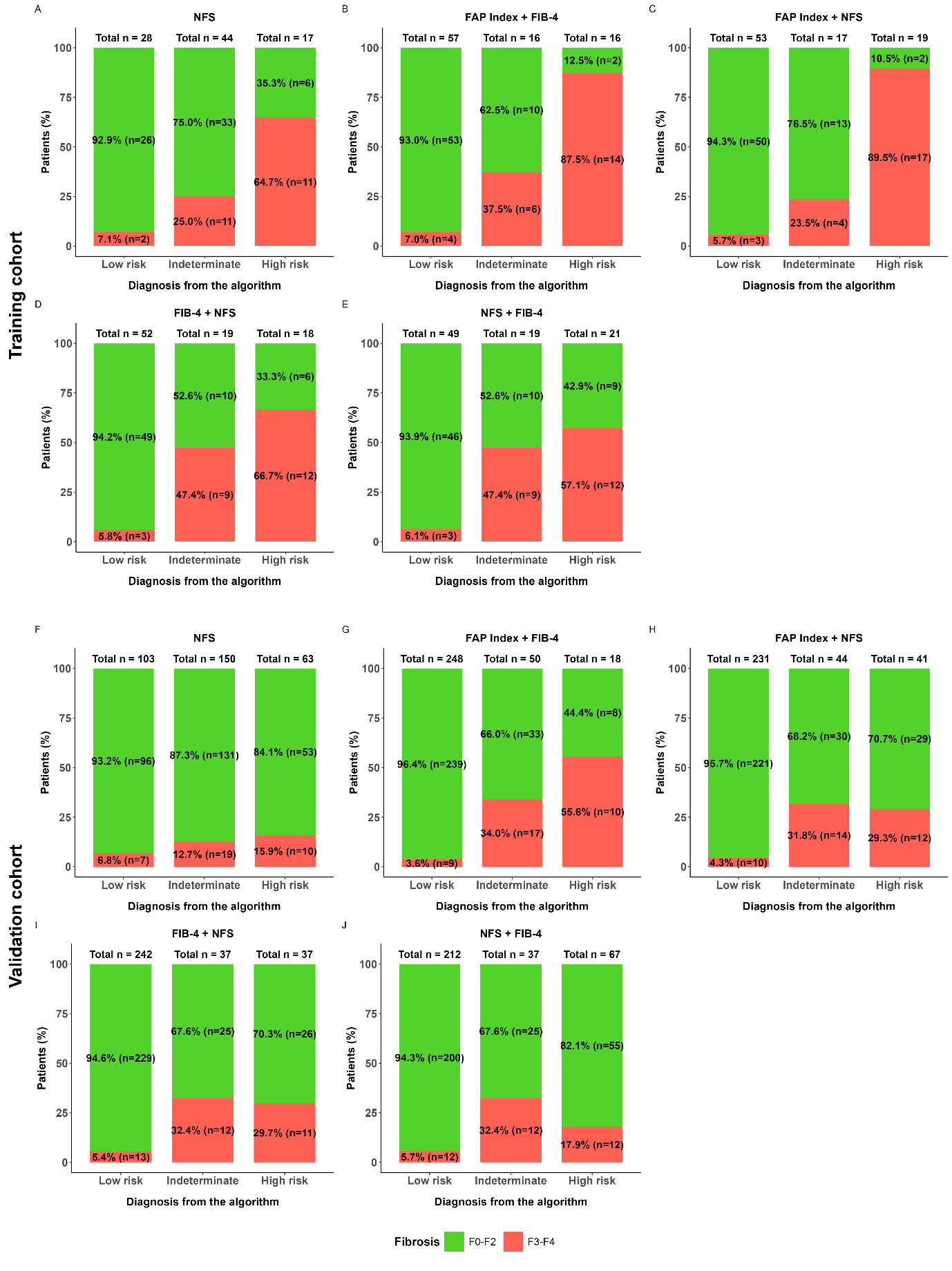


***Supplementary figure 6.*** Fibrosis classification accuracy. Stratification for the risk of advanced fibrosis using the serum-based NITs FAP-Index, FIB4 and NFS, and sequential combinations of these NITs in the training (A-E) and validation (F-J) cohorts. Colours represent F0-F2 (green) and F3-F4 (red) biopsy derived fibrosis score.


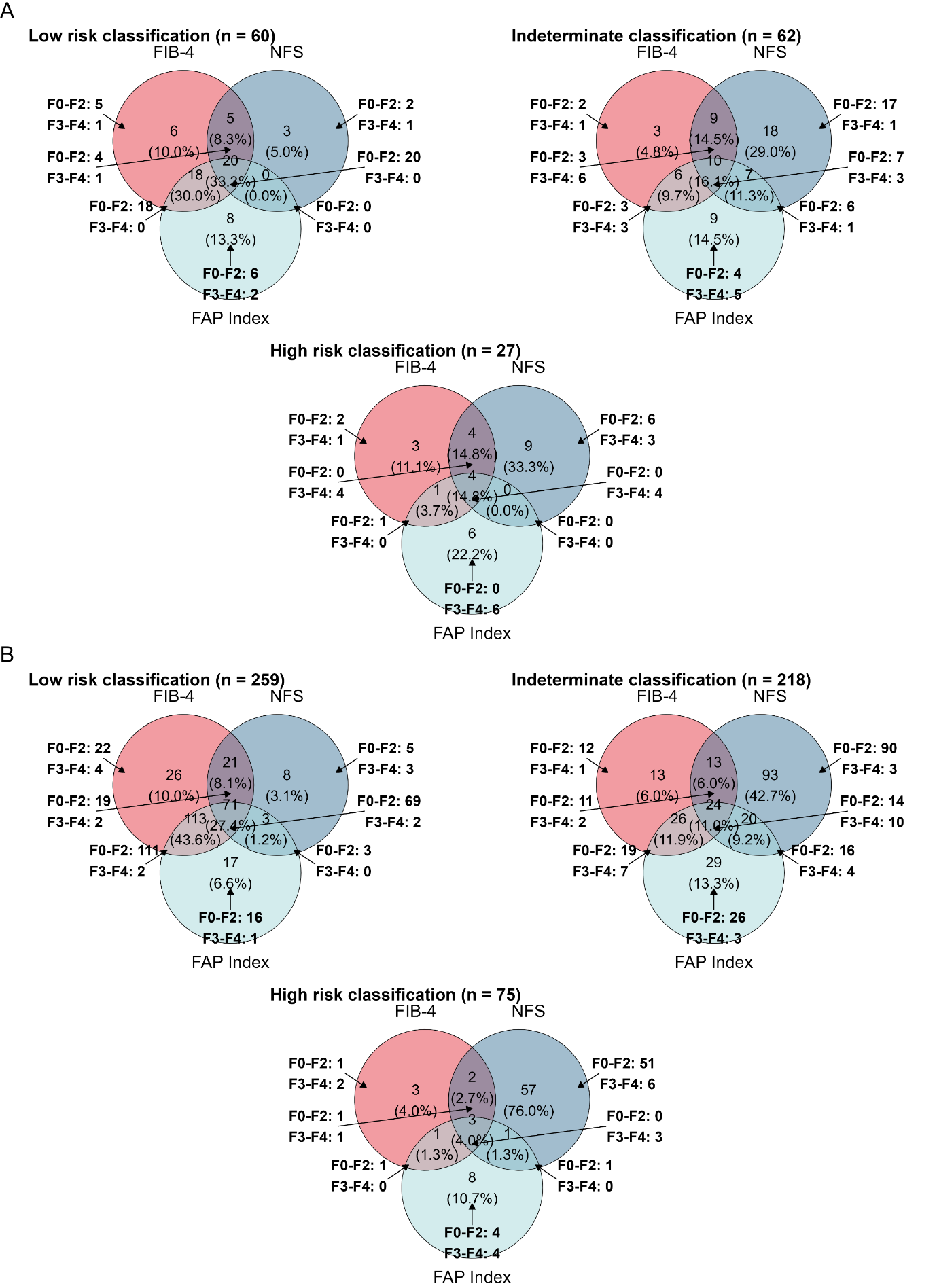


***Supplementary figure 7.*** Venn plots of classification of fibrosis risk as low, indeterminate or high by each algorithm applied to (A) Training cohort, (B) Validation cohort. Each percentage is % of total data in that Venn plot (n).


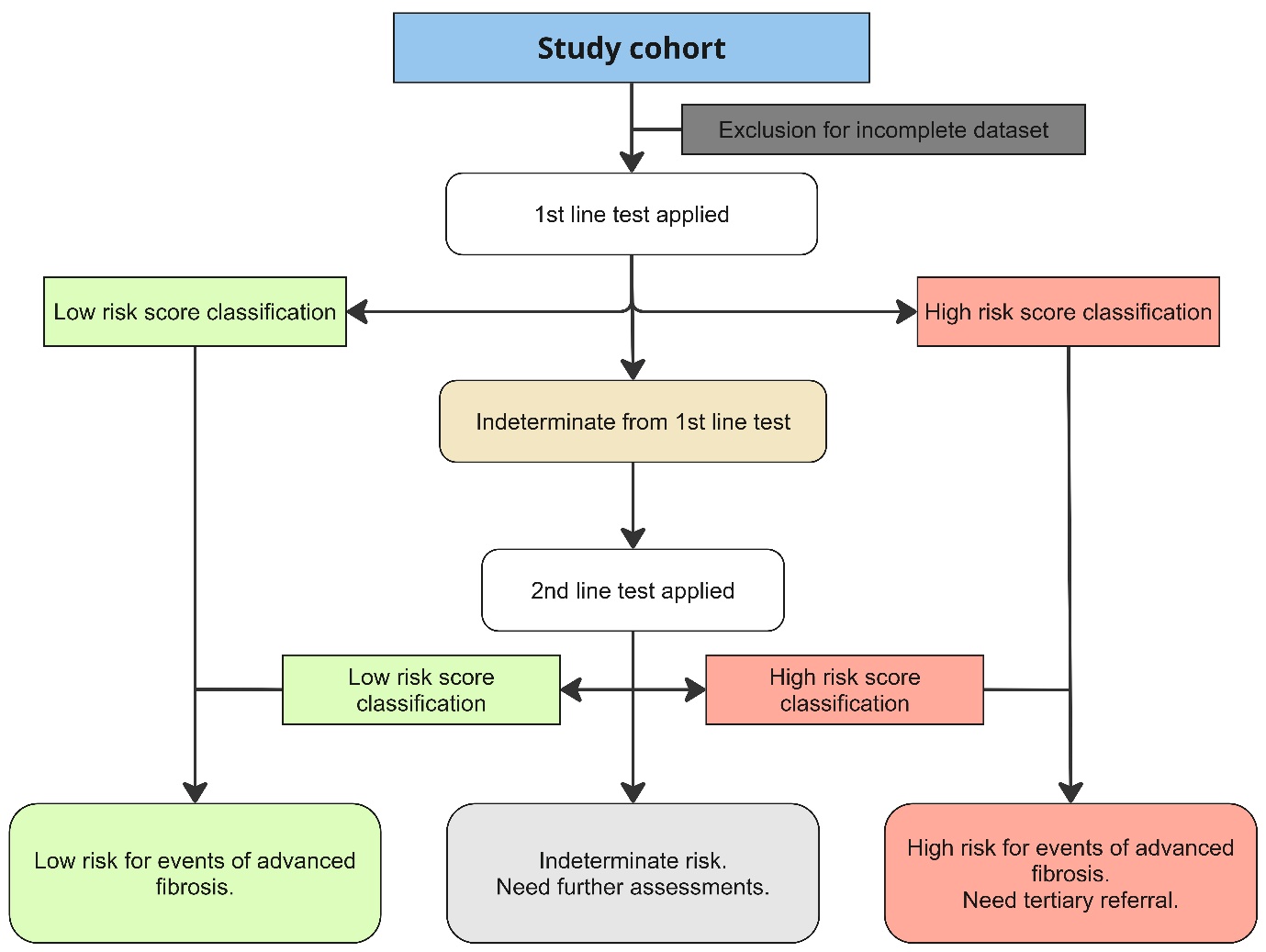


***Supplemental figure 8****.* ***Diagnosis flowchart****.* Generic flowchart of a 2-step sequential application of non-invasive blood biomarker scoring classification.


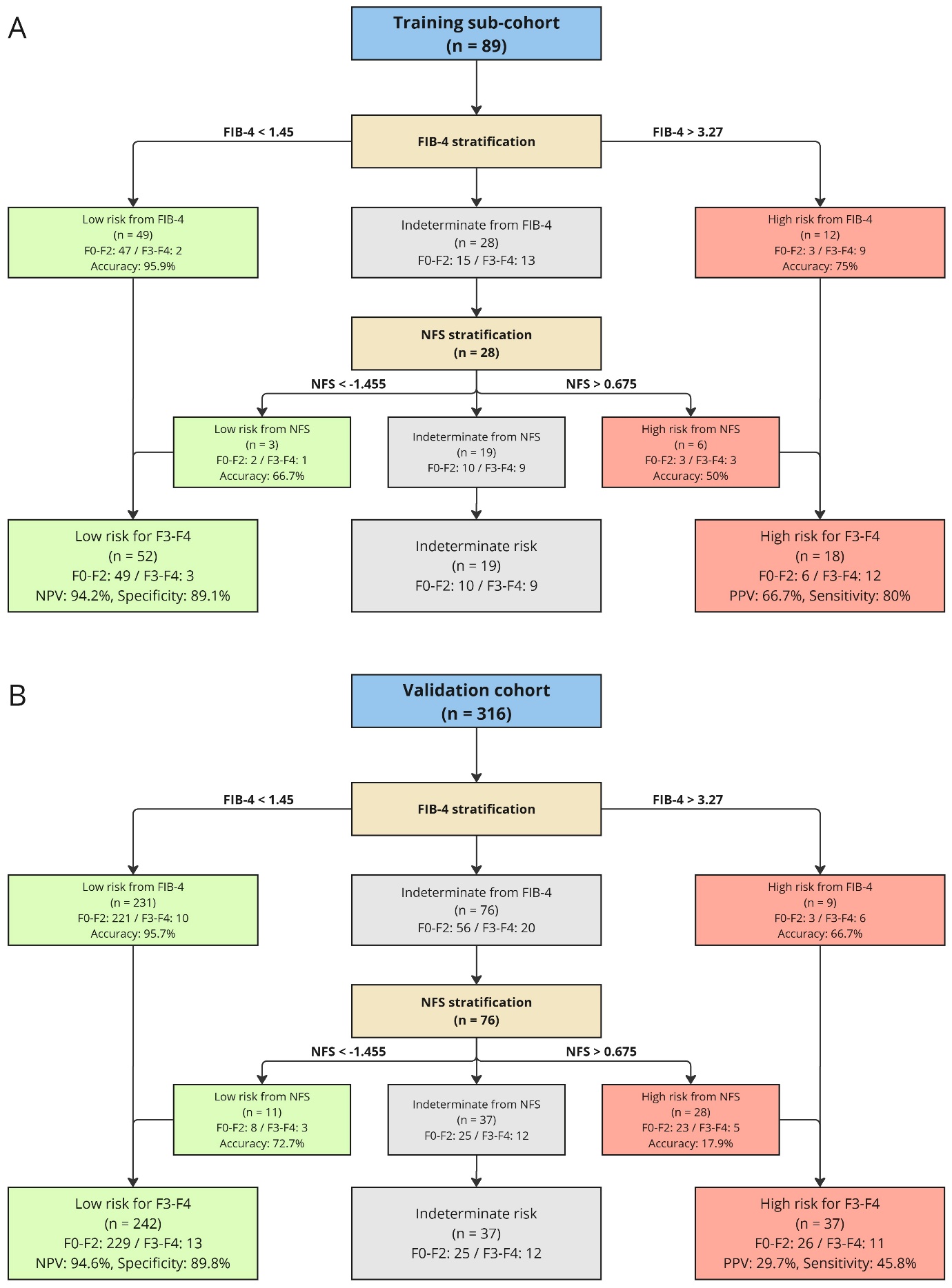


***Supplementary figure 9.*** ***Diagnosis flowchart*.** Flowchart for a risk stratification using two sequential NITs; NAFLD Fibrosis Score (NFS) following FIB-4, to discriminate the presence of advanced fibrosis in (A) training sub-cohort (n=89), (B) validation cohort (n = 316). FIB-4 and NFS cut-offs for indeterminate classification in each algorithm are shown.


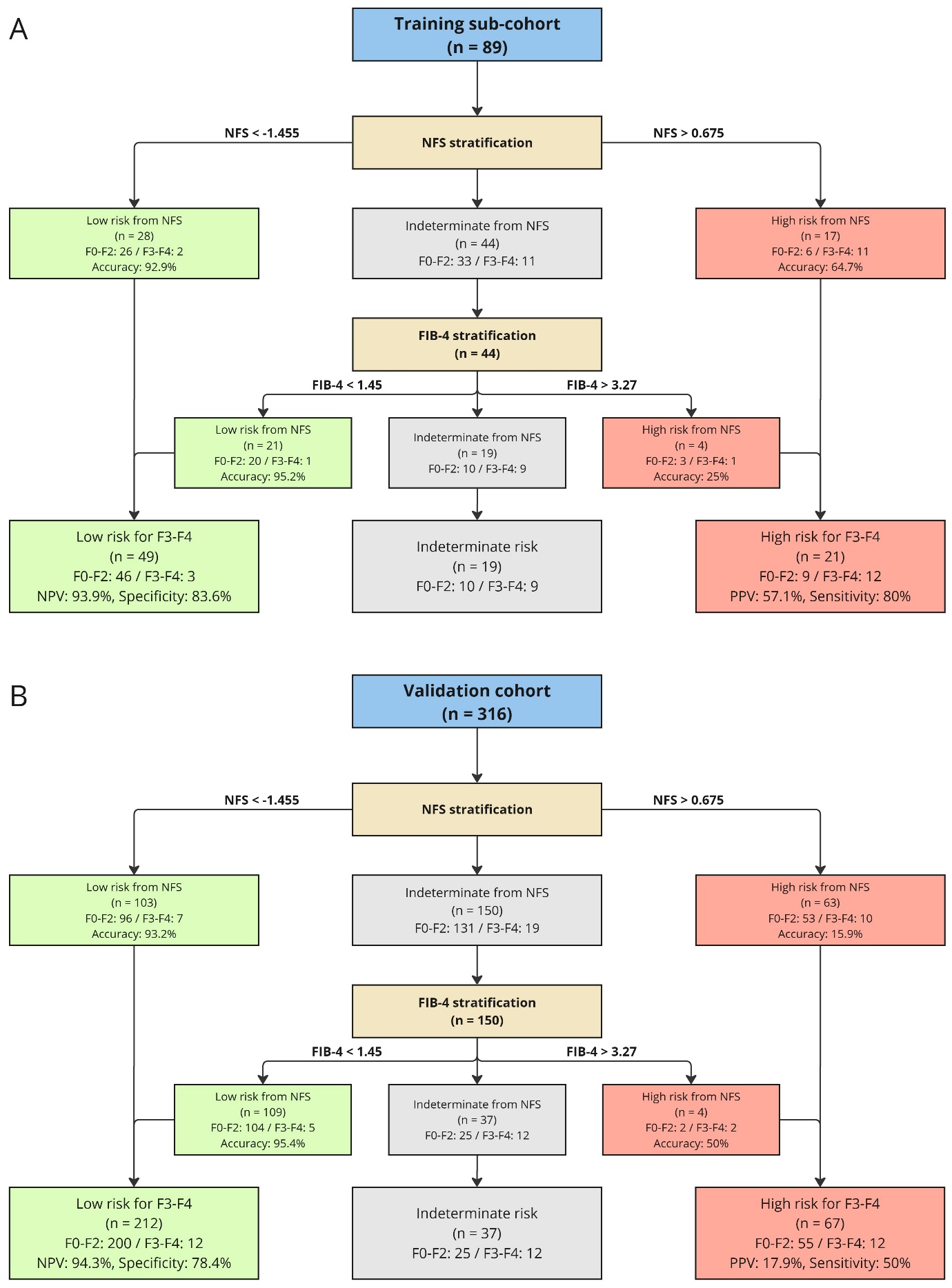


***Supplemental figure 10.*** ***Diagnosis flowchart*.** Flowchart for a risk stratification using two sequential NITs; FIB-4 following NAFLD Fibrosis Score (NFS), to discriminate the presence of advanced fibrosis in (A) training sub-cohort (n=89), (B) validation cohort (n = 316). FIB-4 and NFS cut-offs for indeterminate classification in each algorithm are shown.


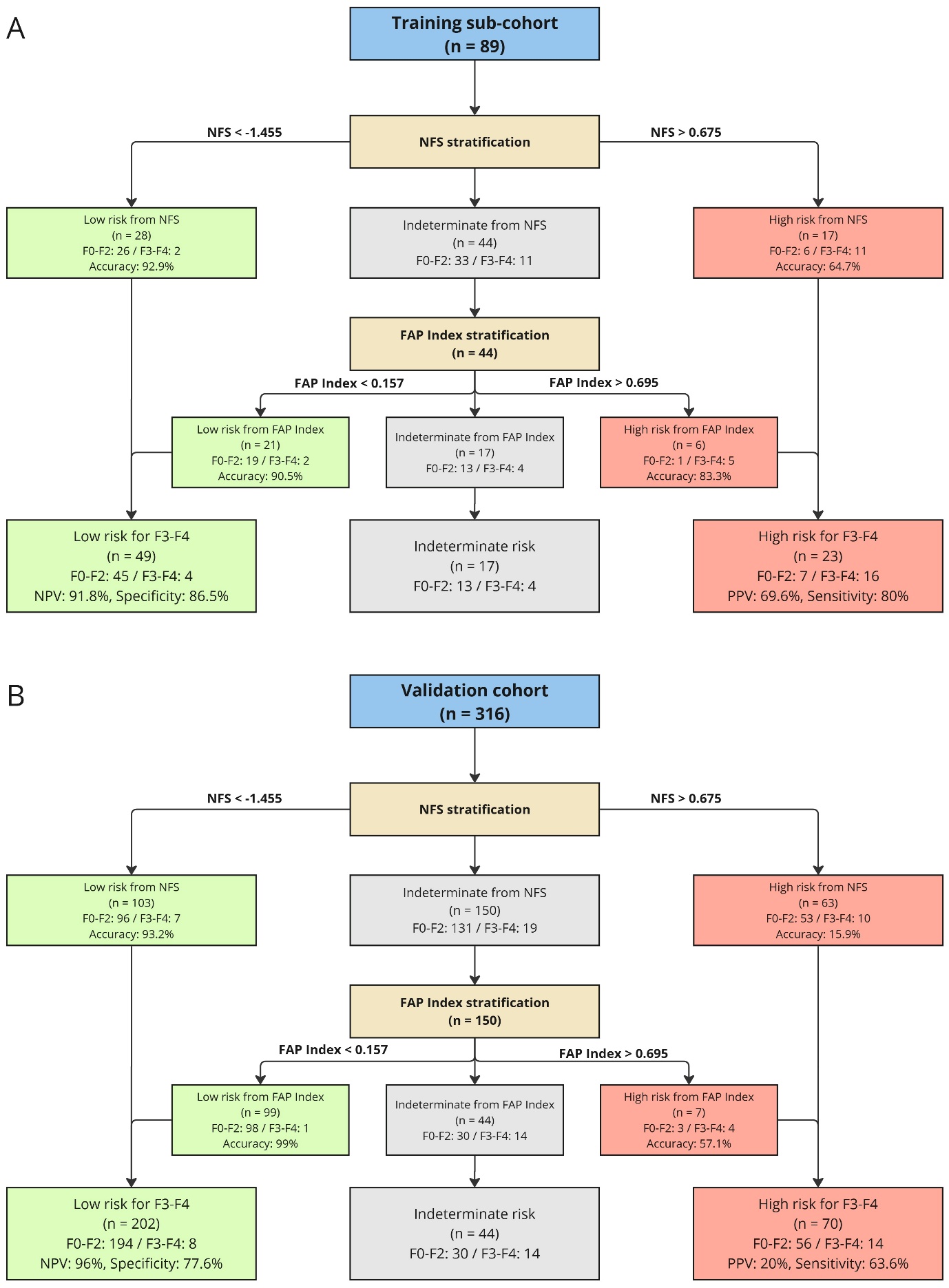


***Supplemental figure 11.*** ***Diagnosis flowchart*.** Flowchart for a risk stratification using two sequential NITs; FAP-Index following NAFLD Fibrosis Score (NFS), to discriminate the presence of advanced fibrosis in (A) training sub-cohort (n=89), (B) validation cohort (n = 316). FIB-4 and FAP-Index cut-offs for indeterminate classification in each algorithm are shown.


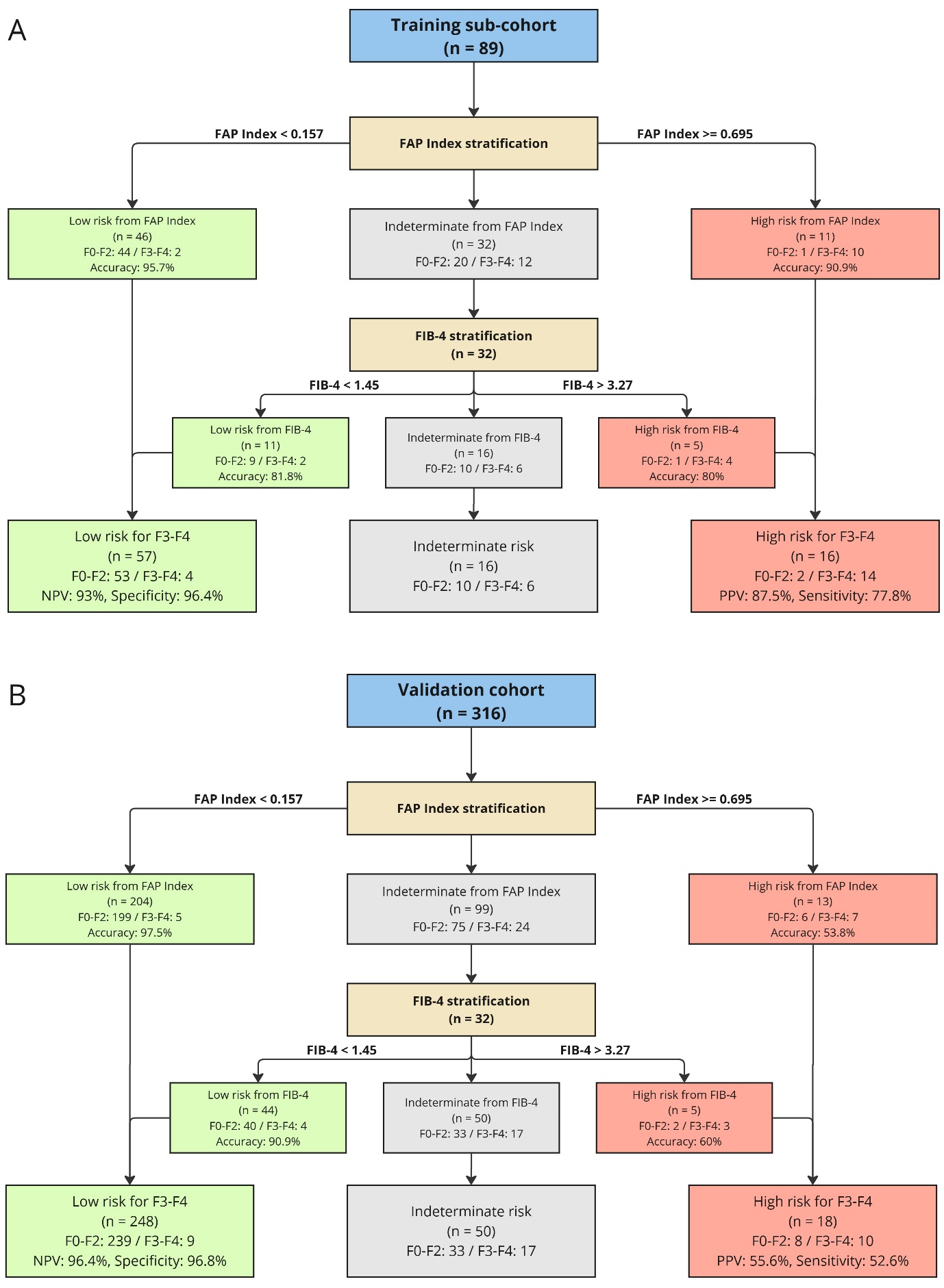


***Supplemental figure 12.*** ***Diagnosis flowchart*.** Flowchart for a risk stratification using two sequential NITs; FIB-4 following FAP-Index, to discriminate the presence of advanced fibrosis in (A) training sub-cohort (n=89), (B) validation cohort (n = 316). FIB-4 and FAP-Index cut-offs for indeterminate classification in each algorithm are shown.


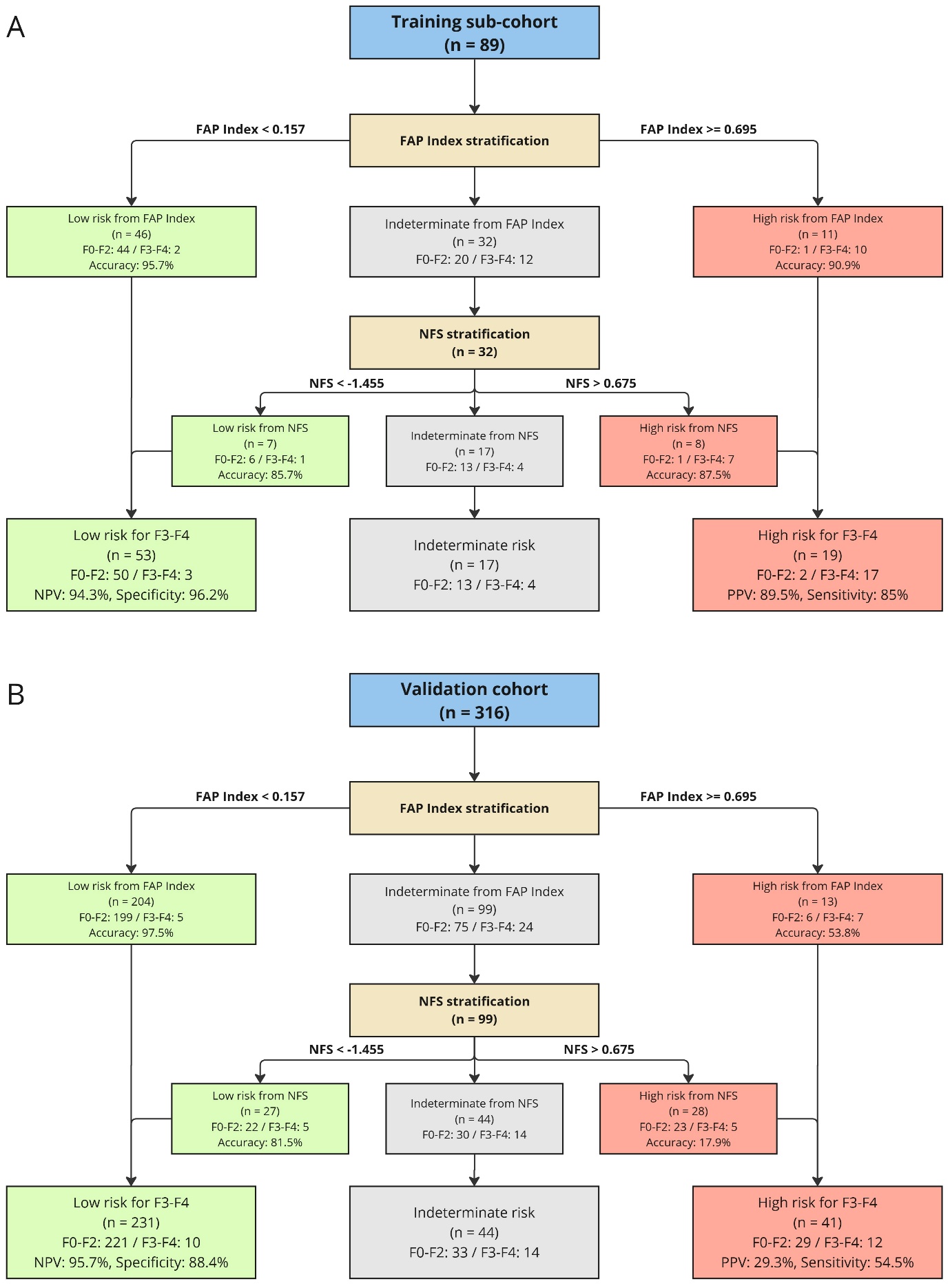


***Supplemental figure 13.*** ***Diagnosis flowchart*.** Flowchart for a risk stratification using two sequential NITs; NAFLD Fibrosis Score (NFS) following FAP-Index, to discriminate the presence of advanced fibrosis in (A) training sub-cohort (n=89), (B) validation cohort (n = 316). FIB-4 and FAP-Index cut-offs for indeterminate classification in each algorithm are shown.


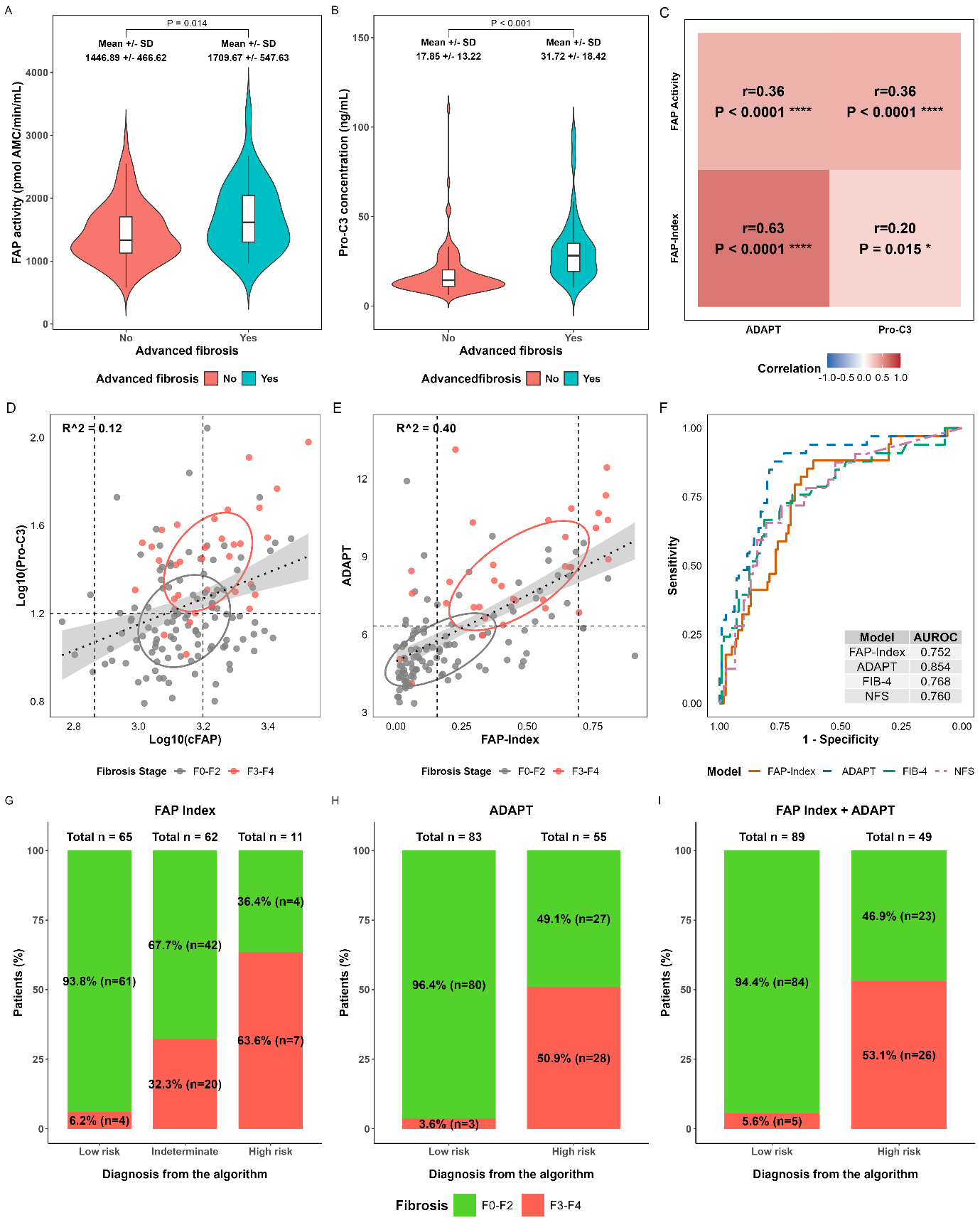


***Supplementary figure 14.*** ***Comparisons of cFAP based FAP-Index with PRO-C3 based ADAPT.*** Evaluation of FAP-related and PRO-C3-related variables for diagnostic potential in identifying advanced fibrosis in cohort W. A, B: Violin plots of (A) cFAP activity and (B) PRO-C3 of patients without (No) and with (Yes) advanced fibrosis. Both cFAP activity and PRO-C3 levels were significantly greater in advanced fibrosis. C: Correlation matrix of cFAP Activity, FAP-Index algorithm, PRO-C3 and ADAPT algorithm, showing the Pearson correlation coefficients (r). D, E: Scatter plots showing relationships between (D) log10-transformed cFAP versus log10-transformed PRO-C3 levels, and (E) ADAPT with FAP-Index, coloured by fibrosis stage, with an ellipse drawn around 50% CI. F: Receiver Operating Characteristic (ROC) for advanced fibrosis using FAP-Index, ADAPT, FIB-4, and NFS. G-I: Stratification for risk of advanced fibrosis using FAP-Index and ADAPT.


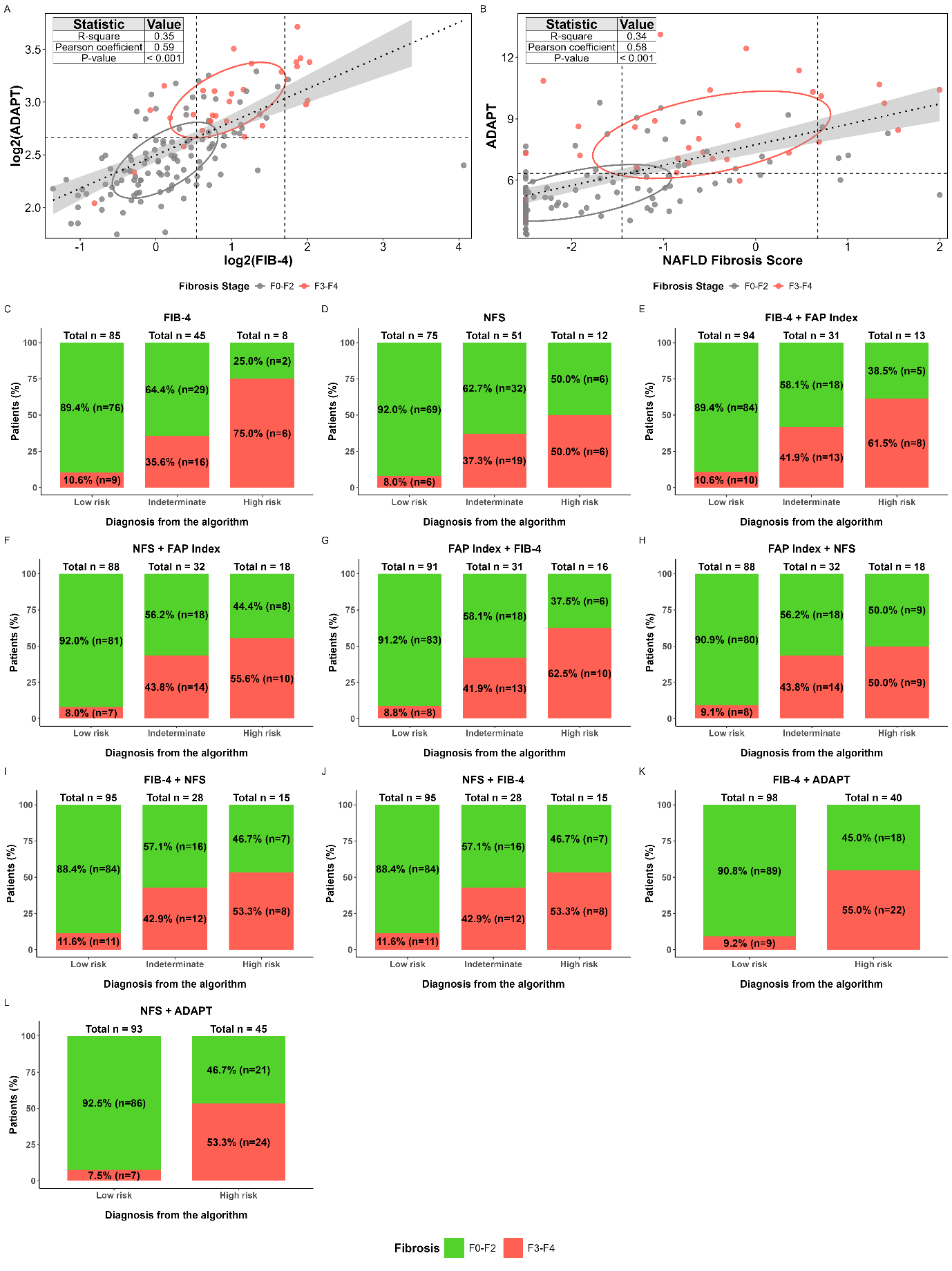


***Supplementary figure 15.*** ***Comparisons of ADAPT with other NITs.*** Scatter plot showing the relationship between (A) log10-transformed FAP-Index and log10-transformed ADAPT, (B) ADAPT algorithm versus NFS, coloured by fibrosis stage, ellipse showing 50% CI. (C-L) Stratification for the risk of advanced fibrosis using serum-based NITs in the training cohort, as an indicator of classification accuracy***.*** F0-F2 (green) and F3-F4 (red) biopsy derived fibrosis score.

**
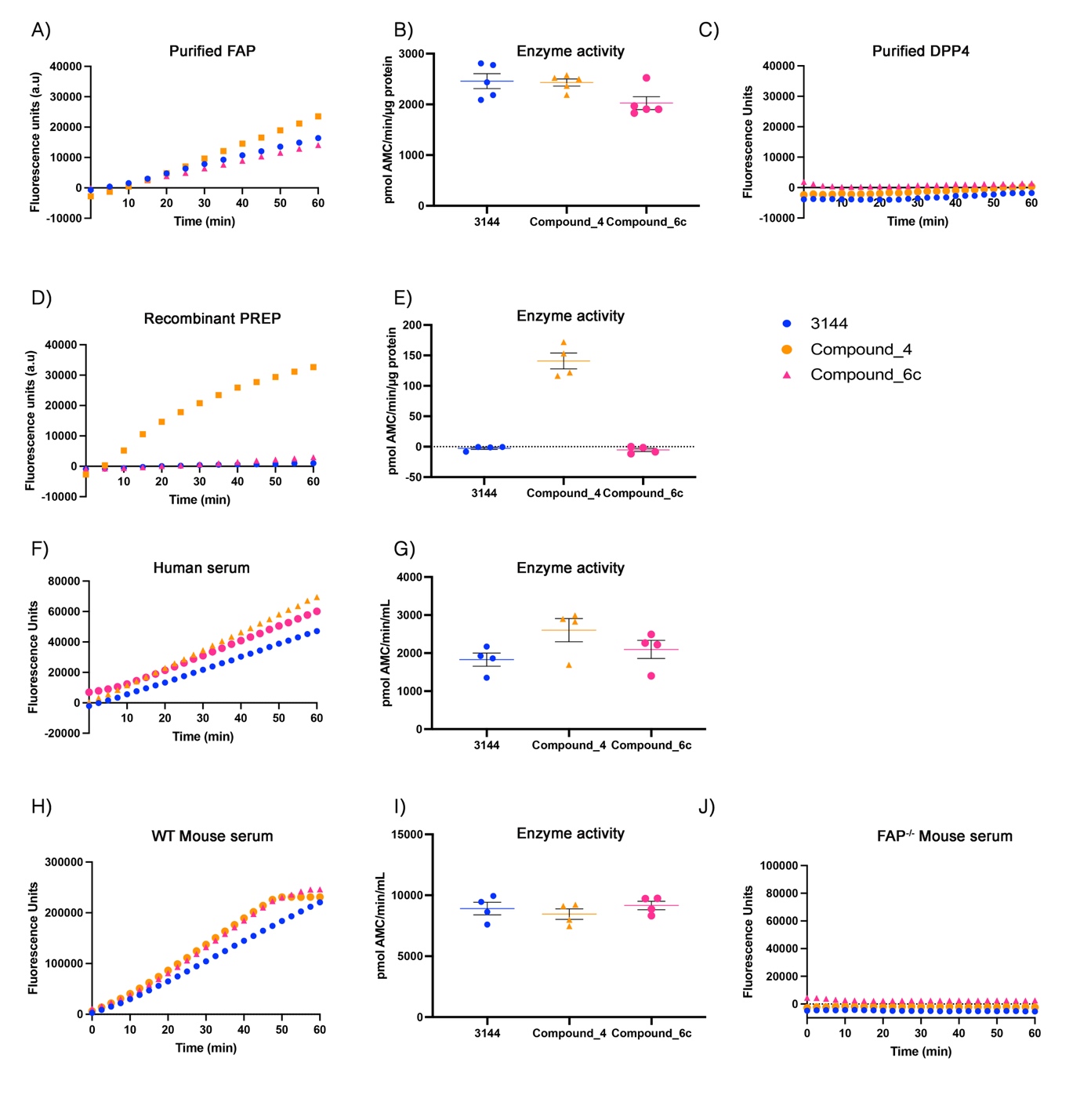
*Supplementary figure 16. 3144-AMC is a specific substrate of fibroblast activation protein alpha (FAP).***

An in-house enzyme-based assay was used to examine hydrolysis by (A) purified FAP, (C) purified DPP4 and (D) recombinant prolyl endopeptidase (PREP) in the presence of 3144-AMC (blue), compound 4 (orange) and compound 6c (pink). Enzyme activity of (B) FAP (n=5) and (E) PREP (n=5) was calculated on the linear segment of each fluorescence plot. All three substrates were each used to determine hydrolysis by human serum (F) and thus enzyme activity (G) (n=5). The three substrates were also used to measure substrate hydrolysis in the serum of wild-type mice (H, I) (n=4) and FAP enzyme negative (FAP-/-) mice (J) (n=4), by published methods[[8](#_ENREF_8)]. These compounds were provided by WWB (3144) and PVDV (compound 4 and compound 6c). Fluorescence was measured every 2.5 minutes for 1 hour at 37 °C in a plate reader with excitation at 355 nm and emission at 450 nm. Mean ± SEM.


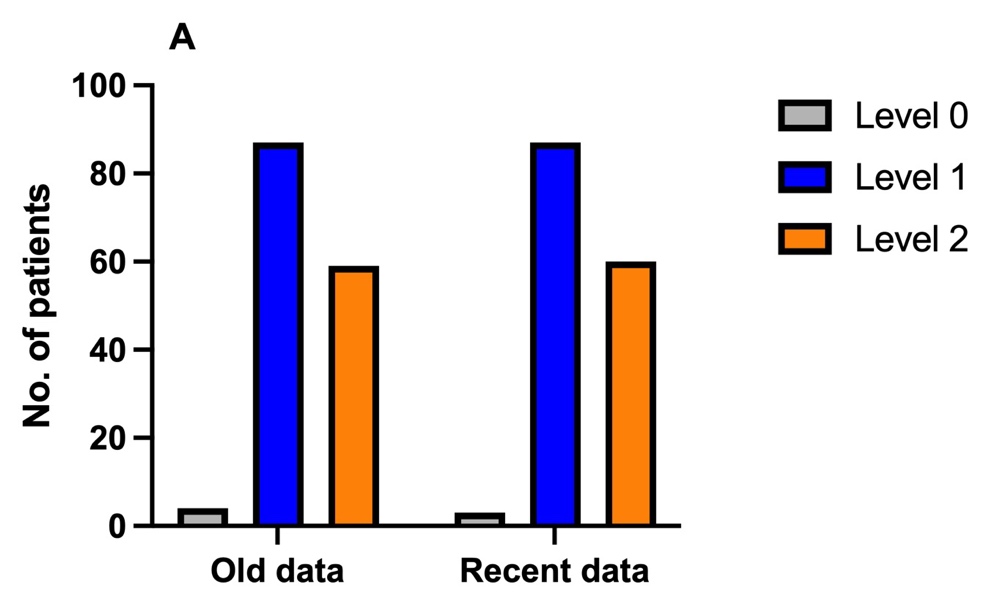


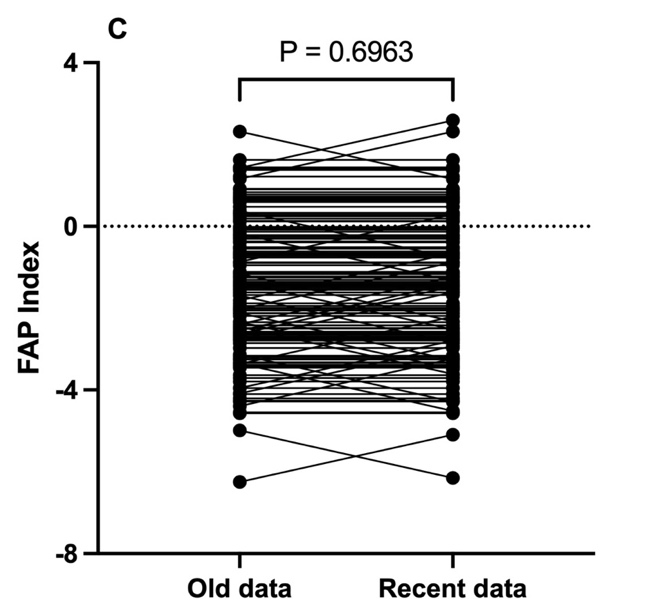

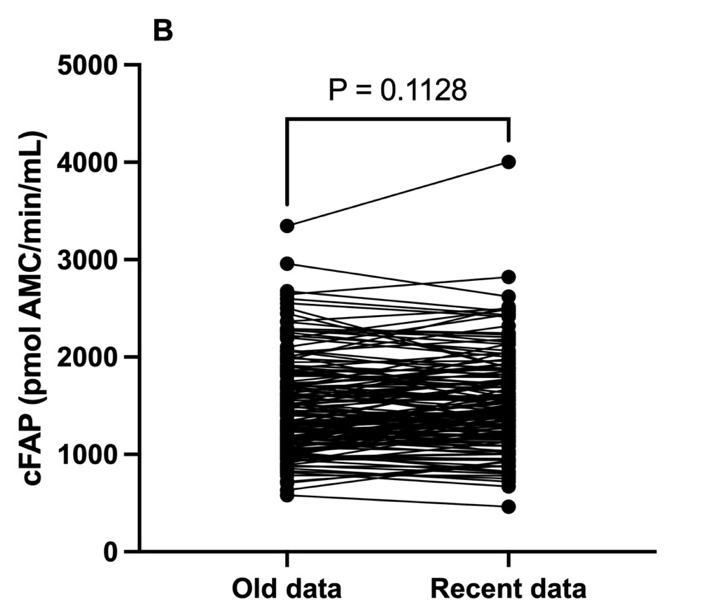


***Supplementary figure 17****.* ***Reproducibility of cFAP measurement by independent operators at different times.*** Pairwise analysis of cFAP activity measured at two different time points and performed by two different persons on serum samples from 150 patients of Westmead Hospital (cohort W). A: The histogram of cFAP activity transformed into the ordinal levels 0, 1 and 2. The independent chi-square test showed no significant difference in the distribution of the ordinal rank (X^2^ = 0.1513, P-value = 0.93). B: Paired t-test showed no significant difference between the two measurements (t_149_ = 1.595, P-value = 0.11). C: Paired t-test showed no significant statistical difference between the two measurements of FAP-Index calculated using the old and new cFAP activity measurements (t_149_ = 0.39, P-value = 0.70).

# References for Supplementary Information

1. Adams LA, Wang Z, Liddle C, et al. Bile acids associate with specific gut microbiota, low-level alcohol consumption and liver fibrosis in patients with non-alcoholic fatty liver disease. Liver international : official journal of the International Association for the Study of the Liver. 2020;40(6):1356-65.

2. Ooi GJ, Earnest A, Kemp WW, et al. Evaluating feasibility and accuracy of non-invasive tests for nonalcoholic fatty liver disease in severe and morbid obesity. Int J Obes (Lond). 2018;42(11):1900-11.

3. Daniels SJ, Leeming DJ, Eslam M, et al. ADAPT: An Algorithm Incorporating PRO-C3 Accurately Identifies Patients With NAFLD and Advanced Fibrosis. Hepatology. 2019;69(3):1075-86.

4. Adams LA, Roberts SK, Strasser SI, et al. Nonalcoholic fatty liver disease burden: Australia, 2019-2030. J Gastroenterol Hepatol. 2020;35(9):1628-35.

5. Roberts SK, Majeed A, Glenister K, et al. Prevalence of non-alcoholic fatty liver disease in regional Victoria: a prospective population-based study. Med J Australia. 2021;215(2):77-82.

6. Eslam M, Newsome PN, Sarin SK, et al. A new definition for metabolic dysfunction-associated fatty liver disease: An international expert consensus statement. J Hepatol. 2020;73(1):202-9.

7. Wallace TM, Levy JC, Matthews DR. Use and abuse of HOMA modeling. Diabetes Care. 2004;27(6):1487-95.

8. Keane FM, Yao TW, Seelk S, et al. Quantitation of fibroblast activation protein (FAP)-specific protease activity in mouse, baboon and human fluids and organs. FEBS Open Bio. 2013;4:43-54.

9. De Decker A, Vliegen G, Van Rompaey D, et al. Novel Small Molecule-Derived, Highly Selective Substrates for Fibroblast Activation Protein (FAP). ACS Med Chem Lett. 2019;10(8):1173-9.

10. Xi CR, Di Fazio A, Nadvi NA, et al. A Novel Purification Procedure for Active Recombinant Human DPP4 and the Inability of DPP4 to Bind SARS-CoV-2. Molecules. 2020;25(22).

11. Xi CR, Di Fazio A, Nadvi NA, et al. An improved production and purification protocol for recombinant soluble human fibroblast activation protein alpha. Protein Expr Purif. 2021;181:105833.

12. Levy MT, McCaughan GW, Marinos G, Gorrell MD. Intrahepatic expression of the hepatic stellate cell marker fibroblast activation protein correlates with the degree of fibrosis in hepatitis C virus infection. Liver. 2002;22(2):93-101.
